# Reprocessable Covalent Adaptable Networks Enabled by Transamidation and Imine Metathesis Exchanges

**DOI:** 10.1021/acspolymersau.6c00150

**Published:** 2026-08-31

**Authors:** Tommaso Telatin, Thijs Van Casteren, Silvia De La Flor, Àngels Serra, Xavier Montané

**Affiliations:** † 16777 Universitat Rovira i Virgili, Department of Analytical and Organic Chemistry, C/Marcel·lí Domingo 1, 43007 Tarragona, Spain; ‡ Universitat Rovira i Virgili, Department of Mechanical Engineering, Av. Països Catalans 26, 43007 Tarragona, Spain

**Keywords:** covalent adaptable networks, imine, amide-imide, adhesive, multiple dynamic covalent bonds

## Abstract

Here, we report the preparation of a series of dynamic covalent poly­(amide-imine) materials synthesized from a biobased triester monomer: methyl tricarballylate (TM), commercial di- and triamines (tris­(2-aminoethyl)­amine, Jeffamine D230, and hexamethylene diamine), and a synthesized imine-containing diamine (IDA). These high-*T*_g_ materials (>79 °C) feature dynamic amide and imine groups that contribute independently to material dynamicity while exhibiting a synergistic effect through transimination reactions promoted by primary amines released during amide dissociation. The coexistence of these two dynamic moieties enhances stress relaxation; however, the relaxation kinetics result from a delicate balance between dynamic chemistry rates and network architecture. We demonstrated that modulating the proportion of the imine-containing monomer, the flow behavior of the prepared materials can be controlled, shifting from a purely dissociative to a mainly associative viscous profile. This acquires great technological relevance considering the critical importance of material flowability in several manufacturing processes and industrial applications. Adhesive testing revealed a strong correlation between adhesive strength and high-temperature viscosity, along with facile debondability under extremely low loads. These findings demonstrate that amide exchange and imine metathesis/transimination are promising dynamic partners for unlocking rheological properties unattainable with conventional thermosets, thereby expanding applications in high-performance fields.

## Graphical abstract

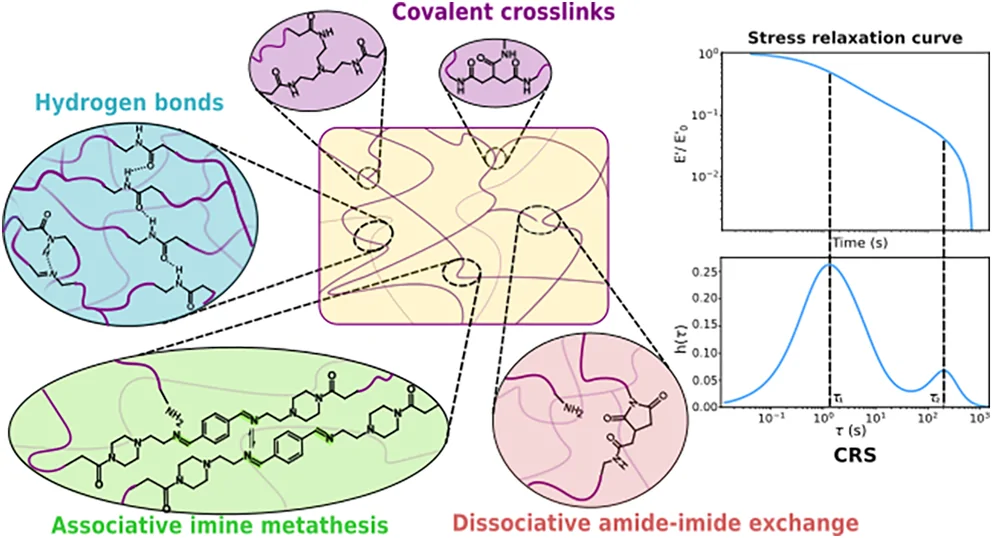

## 1 Introduction

The coexistence of different types of dynamic chemistries is characteristic of many natural systems, where different dynamic bonds act synergistically to enhance the performance of biological structures.^1,2^ Efforts to artificially mimic physiological functions have led to growing interest in how different dynamic systems coexist and influence the properties of the network as well as its responsiveness to specific stimuli.^3^

Polymer networks that host reversible bonds are usually termed dynamic polymer networks (DPNs) and represent a well-established trend in material science. In the specific case where the dynamic moieties consist of covalent bonds capable of exchange reactions, the network is defined as a covalent adaptable network (CAN). Incorporating these kinds of reactive groups makes a wide range of chemical reactions accessible for the development of cross-linked materials, allowing the tuning of a wide range of viscoelastic properties. This approach enables rheological behaviors that are difficult or impossible to achieve with traditional thermosets, making several key manufacturing processes typically used for thermoplastics applicable to cross-linked materials. For instance, Hernandez et al.^4^ studied poly­(styrene-*co*-maleic maleate)-based polymer networks obtained by cross-linking commercially available poly­(styrene-*co*-maleic anhydride) with a series of diols, demonstrating how these materials can be continuously (re)­processed via extrusion and injection molding. The presence of dynamic covalent bonds also confers reshaping capabilities and recyclability to the materials, making them suitable for sustainable applications.^5–7^

A common challenge in the design of CANs is the limited mobility of polymer chains, which can compromise the dynamicity of the system. On the other hand, high chain mobility is often achieved at the expense of mechanical performance, limiting the range of potential applications. From this perspective, the combination of multiple dynamic chemistries within the same network has attracted interest as a strategy to modulate viscoelastic behavior and mechanical performance, enabling access to a diverse range of material properties.^8,9^ Furthermore, noncovalent interactions, such as hydrogen bonding and π–π interactions, have often been exploited to enhance mechanical properties^10–13^ or to accelerate the kinetics of exchange reactions.^14^

The presence of multiple dynamic covalent bonds within the same material provides access to a broader range of strategies for tuning dynamic behavior and rheological properties. One approach consists of improving the dynamic performance by selecting dynamic bonds with different exchange mechanisms that are orthogonal to each other. Du Prez’s group^15^ combined transesterification and siloxane exchange, demonstrating that these two associative exchange reactions act synergistically to facilitate topological rearrangement, resulting in an overall exchange rate faster than the sum of the individual exchange processes.

Particularly effective in unlocking dynamic features hampered by poor chain mobility is the combination of associative and dissociative chemistries. Indeed, dissociation reactions produce partial degelation of the polymer network, reducing the chain hindrance. Therefore, dissociative bonds not only contribute to stress relaxation processes but also alter the microenvironments of the chain segments, reducing the energetic barrier for associative exchange.^16,17^ Different studies^16,18^ have reported dual dynamic networks in which imine metathesis and Diels–Alder reactions are combined, leading to improved reprocessability. Costa Cornellà et al.^19^ combined Diels–Alder and transesterification, finding that tuning the ratio between the chemistries enables control over relaxation dynamics and improves resolution during extrusion processes. Even more interestingly, two well-resolved stress relaxation modes were observed, enabling shape-memory properties that rely solely on the two distinct relaxation dynamics, rather than on the glass transition temperature or melting point.

In this context, transamidation of polyamide has not been widely explored as a partner chemistry in dual dynamic systems. Du Prez and co-workers^20^ first reported the transamidation of polyamides via nucleophilic attack of a neighboring amide on another electrophilic amide group, through a dissociative mechanism involving a cyclic imide intermediate and an amine functionality. The formed imide can then be reopened by another amine to yield a new amide group. The resulting materials exhibited relatively slow stress relaxation processes even at high temperature, in contrast to what was found by Chen et al.,^21^ who proposed a similar dissociative mechanism but obtained significantly higher relaxation rates. In both studies, the materials exhibited very low glass transition temperatures (−10 to 5 °C), which limited their potential applications, particularly in the fabrication of composite materials. From this perspective, our group^22^ reported a system in which poly­(amido-imide) networks were obtained from methyl tricarballylate and commercially available amines, such as 1,6-hexamethylene diamine (HMDA) and tris­(2-aminoethyl)­amine (TREN). In this case, *T*_g_ *s* > 90 °C were obtained, and the kinetics of the exchange process were significantly enhanced by adding different lanthanide triflates as catalysts without compromising creep resistance.

Building on our previous system, this work adopts an alternative strategy that couples the transamidation of polyamide, mediated by neighboring group participation, with imines serving as the second dynamic group. We synthesized an imine-containing secondary diamine monomer (IDA) and employed it in different proportions, while a polyether diamine (Jeffamine D230) was used as a spacer to mitigate the rigidity of the IDA monomer. We thoroughly investigated the effect of the network architecture on the thermomechanical properties, the kinetics of network rearrangement, and the synergistic effects arising from the coexistence of the two dynamic systems within the same network.

## 2 Experimental Section

### 2.1 Materials

Tricarballylic acid (98%, TCA), anhydrous magnesium sulfate (MgSO_4_), sodium carbonate (Na_2_CO_3_), and tris­(2-aminoethyl)­amine (97%, TREN) were purchased from Thermo Scientific. Sulfuric acid (95%, H_2_SO_4_), diethyl ether (Et_2_O), and deuterated chloroform (CDCl_3_, 99.8% D) were purchased from VWR Chemicals. Terephthalaldehyde (99%, TA), poly­(propylene glycol) bis­(2-aminopropyl ether) (*M_n_* ∼ 230, Jeffamine D230), hexamethylene diamine (98%, HMDA), 2-aminoethyl piperazine (99%, AEP), sodium silicate solution (Na_2_SiO_3_), sodium phosphate (96%, Na_3_PO_4_), and sodium dodecylbenzenesulfonate (technical grade, SDBS) were purchased from Sigma-Aldrich, while tetrahydrofuran (THF), methanol (MeOH), and *n*-butanol (*n*-BuOH) were obtained from Scharlau. All chemicals were used as received. Methyl tricarballylate (MT) and the imine-containing secondary diamine (IDA) were synthesized as described in the Supporting Information (Schemes S1 and S2, respectively). The ^1^H NMR spectra of MT and IDA are shown in Figures S1 and S2.

### 2.2 Preparation of Poly­(amide-imine) Dynamic Networks

Methyl tricarballylate (MT) was weighed and mixed with various proportions of the secondary diamine containing two imine groups (IDA), TREN, and Jeffamine D230, or HMDA, in a 10 mL vial equipped with a magnetic stirrer. The composition of the formulations is detailed in Table 1. 5 mL of *n*-BuOH was added to facilitate the dissolution of IDA. The mixture was then heated to 90 °C and stirred until complete homogenization. The material formation was conducted in a conventional oven at 140 °C for 12 h; then, the material was ground, and an additional 4 h at 140 °C were performed. Finally, the materials obtained were hot pressed at 140 °C for 30 min under 1.2 t (2.3 MPa). The materials were encoded as PAI-X-Y, where PAI stands for poly­(amide-imine), X refers to the primary diamine used (either Jeffamine D230 or HMDA), and Y represents the proportion of secondary amine equivalents from IDA relative to the ester equivalents from MT.

**Table 1:** Monomer Composition and Concentrations of PPO Spacer Units and Imine Groups in the Prepared Materials

sample	MT (mol)	IDA (mol)	TREN (mol)	D230 (mol)	HMDA (mol)	[PPO]^a^ (mmol/g)	[imine]^b^ (mmol/g)
PAI-D230–0.25	10.000	3.750	3.750	5.625		1.28	1.70
PAI-D230–0.50	10.000	7.500	2.500	3.750		0.73	2.93
PAI-D230–0.75	10.000	11.250	1.250	1.875		0.32	3.85
PAI-HMDA-0.25	10.000	3.750	3.750		5.625		1.99

a The concentration of PPO units was calculated from the proportion of Jeffamine D230.

b The concentration of imine groups was calculated from the proportion of IDA.

### 2.3 Characterization Techniques

Proton nuclear magnetic resonance (^1^H NMR) spectra were recorded in a Varian VNMR-S400 NMR spectrometer (^1^H–400 MHz). CDCl_3_ was used as the solvent. All chemical shifts are quoted on the δ scale in parts per million (ppm) using residual protonated solvent signal as the internal standard (^1^H NMR: CDCl_3_ = 7.26 ppm).

A Jasco FT/IR-680 Plus spectrometer equipped with a temperature-controlled attenuated total reflection (ATR) accessory (Golden Gate, Specac Ltd., Teknokroma) was used to record the Fourier transform infrared (FTIR) spectra of the fully cured materials. Real-time spectra were recorded in absorbance mode over a wavenumber range of 4000 and 600 cm^–1^, with a resolution of 4 cm^–1^, averaging 64 scans for each spectrum. Background was registered at each temperature, and each spectrum was acquired after an equilibration time of 5 min.

The thermal stability of the materials was evaluated using a Mettler Toledo TGA 2 thermobalance. Cured samples, weighing around 10 mg, were degraded between 30 and 600 °C at a heating rate of 10 °C min^–1^ in an N_2_ atmosphere with a flow rate of 50 cm^3^ min^–1^.

The thermomechanical properties were studied using a DMTA Q850 (TA Instruments) equipped with a film tension clamp. Prismatic rectangular samples with dimensions of around 15 × 4 × 0.3 mm^3^ were analyzed from – 20 to 200 °C at 1 Hz, with 0.1% strain at a heating rate of 2 °C min^–1^.

DSC analyses were performed on a Mettler DSC3+ instrument calibrated using indium (heat flow calibration) and zinc (temperature calibration) standards. Samples of approximately 5–10 mg were placed in aluminum pans with pierced lids and analyzed in an N_2_ atmosphere with a gas flow of 50 cm^3^ min^–1^. Glass transition temperatures (*T*_g_) were determined under dynamic scans between −5 and 250 °C at a heating rate of 10 °C min^–1^.

On stress relaxation measurements, the relaxation modulus (*E*(*t*,*T*)) was followed over time at constant temperature using a constant tensile strain of 1%, within the linear viscoelastic region of the samples. All series of stress relaxation experiments at different temperatures were performed successively on the same sample, always starting the set at the highest indicated temperature. Creep experiments were carried out using an ARES-G2 rheometer (TA Instruments). All of the tests were performed in parallel plate geometry using 25 mm sample disks, using a normal force of 3 N while applying a constant stress τ of 2 kPa, monitoring the gap between each plate. Prior to each experiment, the samples were annealed at 180 °C for 5 min and then cooled down to 110 °C to remove their thermal history. For each single creep measurement, creep compliance (*J*(*t*,*T*) = γ­(*t*,*T*)/τ) was followed for 60 min at each temperature. All creep measurements were conducted successively on the same sample, starting at the lowest temperatures.

### 2.4 Solubility Tests

Samples with a weight of around 40–50 mg were refluxed in 20 mL of THF for 24 h. After cooling, the samples were filtered and vacuum-dried at 70 °C overnight. The gel content was calculated as follows

1 $$\mathrm{gel}\;\mathrm{content}\;\left(\%\right)=\frac{m_{d}}{m_{i}}\cdot 100$$

where *m*_*i*_ and *m*_*d*_ are the initial and the dried masses of the sample, respectively.

### 2.5 Adhesion Tests

Lap shear tests were conducted at room temperature using an electromechanical universal testing machine (Shimadzu AGS-X) with a 10 kN load cell at 10 mm min^–1^ to explore the mechanical properties.

Lap shear tests were carried out using aluminum platens 6060 T66 of dimensions 100 × 25 × 2 mm^2^, according to UNE-EN ISO 1465:2009 standard.^23^ The aluminum platen’s surface was prepared according to UNE-EN ISO 13887:2004 standard.^24^ Overlapping regions were mechanically abrased with P180 sandpaper and treated with an alkaline degreasing aqueous solution of the following composition: 1% Na_2_SiO_3_, 1% Na_2_CO_3_, 1.5% Na_3_PO_4_, 0.5% SDBS as established by Surós et al.^25^ Substrates were immersed in the solution at 80 °C for 10 min, washed with deionized water, air-dried for 10 min, and then dried in an oven for 10 min at a maximum temperature of 70 °C. After that, the precured material of rectangular shape of around 25 × 12.5 × 0.2 mm^2^ was placed in the overlapping region, applying 6 kPa of constant pressure to ensure good contact between the adhesive and the adherents, and finally heated at 160 °C for 1 h.

## 3 Results and Discussion

### 3.1 Design of the Dual Dynamic Networks

In the system previously studied by Roig et al., poly­(amido-imide) networks were prepared by bulk aminolysis reaction of tricarballylic ester (MT) with a mixture of a triamine (TREN) and a diamine (HMDA) in different proportions.^22^ In the present study, HMDA was partially substituted with an imine-containing secondary diamine monomer (IDA) to introduce a second type of dynamic bond into the network. In this way, dissociative amide exchange and associative imine metathesis (Figure 1A,B) reactions coexist in the same polymeric matrix. Amide exchange occurs by means of the nucleophilic attack of a neighboring amide on another electrophilic amide, forming a cyclic imide and releasing a primary amine (Figure 1A). This mechanism determines the presence of free amines in the network when cyclization is triggered by temperature, potentially promoting transimination reactions with the existing imines (Figure 1C). It is important to note that tertiary amides, derived from the reaction of secondary amines from IDA, do not undergo amide exchange.

**Figure 1:**
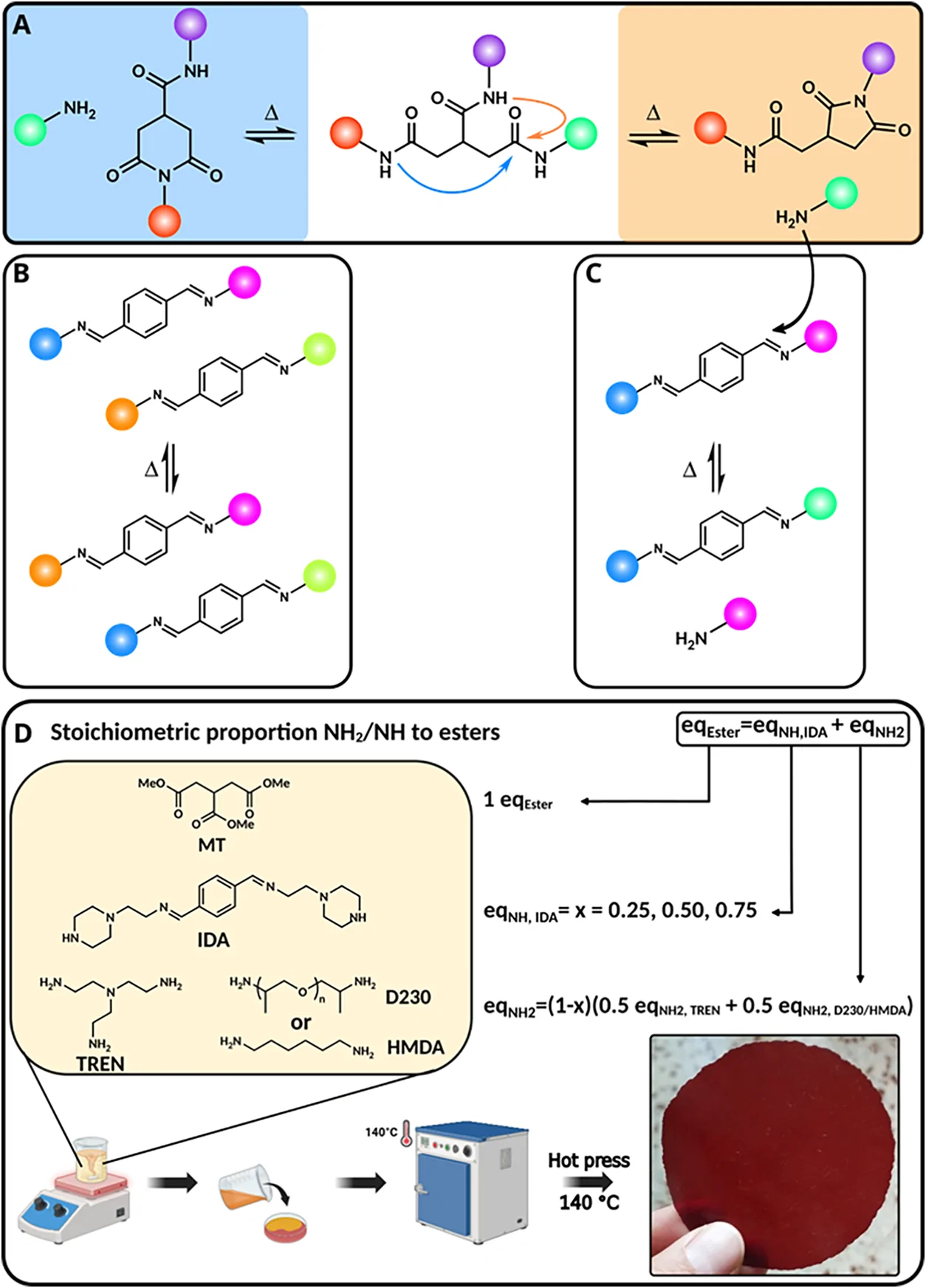
Dynamic reactions that can occur in the prepared PAI networks: (A) dissociation mechanism of neighboring amides by formation of five- or six-membered ring imides using tricarballylic-based structures; (B) metathesis reaction in terephtalaldehyde-derived imine groups; (C) possible transimination reaction made possible by the release of primary amines during amide dissociation; and (D) schematic illustration of the preparation of the materials.

IDA was synthesized by imine condensation as described in the SI (Section S1), resulting in an orange solid that was difficult to dissolve in the reaction mixture. The addition of a high-boiling solvent, such as *n*-butanol, to the reactive mixture was essential to allow the monomers to homogenize. Moreover, due to the high rigidity imparted to the network by the aromatic ring of IDA, a polyether diamine such as Jeffamine D230 was used instead of HMDA to reduce the cross-link density and increase the chain mobility. As shown in Figure 1D, different proportions of IDA were used in the preparation of the poly­(amide-imine) dynamic networks (PAI), while equal equivalents of TREN and D230 were added, reaching a stoichiometric proportion between amine and ester groups. Table 1 summarizes the composition of each prepared material.

### 3.2 Structural Characterization of the Poly­(amide-imine) Dynamic Networks

FTIR analysis was first employed to investigate the network structure of the obtained materials. Figure 2 shows the FTIR spectra of the PAI-D230 materials.

**Figure 2:**
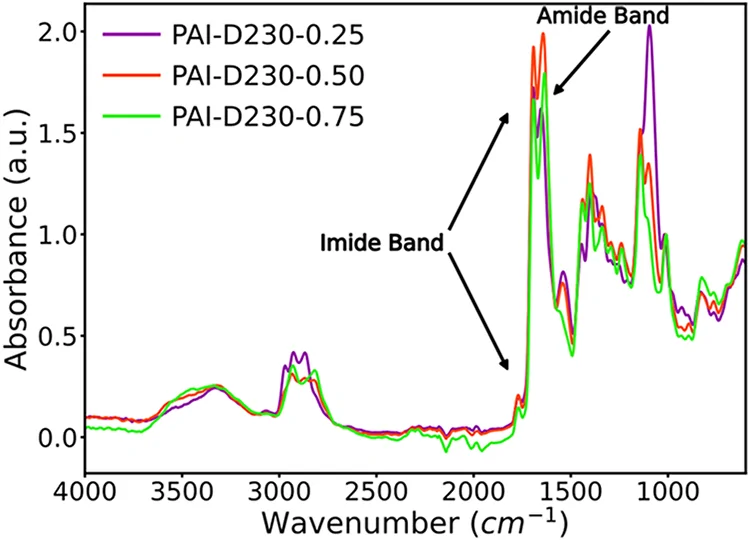
FTIR spectra of the PAI-D230 cured materials at 30 °C.

As can be seen from these spectra, the ester band at 1724 cm^–1^ relative to the MT monomer has completely vanished, while a new band at 1646 cm^–1^ corresponding to the amide group has appeared, confirming the amidation reaction. Although a complete reaction with MT was theoretically possible, some imides were formed during curing, as indicated by the new bands that appear at 1750 and 1696 cm^–1^, respectively. The formation of these imides during curing is a thermodynamically controlled process in which the curing temperature shifts the equilibrium toward the imide product. The cooling procedure produced a “back-shift” of the equilibrium toward the amide, but once this equilibrium was reached, the glass transition topologically locked the network conformation, impeding further evolution of the equilibrium at lower temperatures. According to the literature,^20,22^ this is common for this type of reaction, but it leads to a stoichiometric imbalance in the material, which may affect the integrity of the network and the thermomechanical properties of the materials. The formation of imides results in the presence of free amine groups in the material. If these are primary, they can lead to transamination reactions.

Solubility tests were performed to verify that the imide formation during curing did not compromise the three-dimensional network architecture. All of the materials showed a gel content higher than 96% (Table 2), confirming a generally good network connectivity.

**Table 2:** Gel Content and Main Thermal and Thermomechanical Data Extracted from TGA and DMA Experiments, Respectively

sample	gel content^a^ (%)	*T*_1%_ ^b^ (°C)	*T*_max_ ^c^ (°C)	char yield^d^ (%)	*T*_Loss_ ^e^ (°C)	*E*′_Glassy_ ^f^ (MPa)	*E*′_Rubbery_ ^g^ (MPa)	*T*_tanδ_ ^h^ (°C)	*T*_*g*_ ^i^ (°C)
PAI-D230–0.25	96	244.9	399.1	24.4	32	2845	2	75	79.6
PAI-D230–0.50	97	242.9	401.8	25.5	52	3251	7	117	74.0
PAI-D230–0.75	97	246.7	397.1	28.6	78	2832	3	129	81.7
PAI-HMDA-0.25	98	246.6	399.0	21.6	65	4001	10	96	76.0

a Determined after soaking the material in THF at reflux for 24 h and subsequently vacuum-dried at 70 °C overnight.

b Temperature of 1% weight loss.

c Temperature of the Maximum Degradation Rate.

d Char residue at 600 °C.

e Temperature at the maximum of the *E*″ peak at 1 Hz.

f Glassy storage modulus at *T*_*g*_ – 50 °C determined by DMA.

g Rubbery storage modulus at *T*_*g*_ + 50 °C determined by DMA.

h Temperature at the maximum of the tan δ peak at 1 Hz.

i Glass transition temperature determined by DSC at 10 °C min^–1^.

### 3.3 Thermal and Thermomechanical Properties

The thermal stability of the different PAI materials was assessed by TGA conducted under an N_2_ atmosphere. Figure S3 displays the TGA and DTGA curves, respectively, illustrating the weight loss and its rate versus temperature for all of the prepared materials. The main data are summarized in Table 2.

All of the materials possess high thermal stability (*T*_1%_ > 240 °C), which ensures the feasibility of relaxation, reshaping, or recycling at temperatures below 200 °C. Changing the IDA content does not significantly affect the degradation pattern, as demonstrated by similar *T*_max_ values, while the char yield increases due to the aromatic character of the imine-containing monomer.

The thermomechanical properties of the prepared materials were investigated by DMA. Figure S4A–D presents the evolution of storage modulus, loss modulus, and *tanδ* with temperature for all of the PAI materials. The main data obtained from these experiments are collected in Table 2. The content of D230 strongly affects the viscoelastic response of the materials, determining the concentration of polypropylene oxide units in the network ([PPO] in Table 1). Acting as a spacer, PPO enhances chain mobility, shifting the onset temperature of short-range segmental motion, generally revealed by the peak of *E*″, to lower temperatures. Increasing the IDA content implied a smaller proportion of D230, moving *T*_Loss_ to higher temperatures. Even in this case, the densely cross-linked network of PAI-HMDA-0.25 exhibits lower short-range mobility than PAI-D230–0.25.

The storage modulus in the glassy state (*E*′_Glassy_) is affected by several factors. On the one hand, increasing the content of IDA in the PAI-D230 series increases the number of aromatic rings, which confers superior rigidity to the network due to the formation of π–π interactions. On the other hand, IDA reacts with the ester groups of MT to form tertiary amides. Unlike the secondary amides formed from the reaction of primary amines from TREN, HMDA, and D230, these tertiary amides are not involved in the formation of hydrogen bonds or imide rings. For these reasons, PAI-D230–0.25 and PAI-D230–0.75 exhibit similar *E*′_Glassy_ values despite having very different IDA content. However, while in the former case, the modulus value is mainly ascribable to hydrogen bonding, in the latter case, π–π interactions dominate the physical interactions in the network. In PAI-D230–0.50, a comparable concentration of secondary and tertiary amides results in the presence of both types of interactions, leading to the highest *E*′_Glassy_ value in the PAI-D230 series. Finally, replacing D230 with the shorter HMDA in PAI-HMDA-0.25 produces a more hindered network, which results in a higher modulus.

Increasing the IDA content from 25 to 50% significantly increases *E*′_Rubbery_, from 2 to 7 MPa, despite the lower content of trifunctional TREN in PAI-D230–0.50. From this point of view, the lower concentration of polypropylene oxide units ([PPO]), which are replaced by the shorter, more rigid IDA units, leads to the formation of a more densely cross-linked network. The critical effect of PPO spacers in the network is further confirmed by comparing PAI-D230–0.25 with PAI-HMDA-0.25, which shows how the substitution of D230 with the shorter HMDA results in an *E*′_Rubbery_ value that is five times higher. Intermolecular hydrogen bonds also play a crucial role in modulating *E*′_Rubbery_ by acting as physical cross-links. In PAI-D230–0.50, the higher proportion of IDA lowers the concentration of secondary amides, thereby reducing the number of protons prone to H-bonding. At the same time, the higher content of imine groups increases the number of nitrogen atoms available as H-bond acceptors, which enhances the overall probability that an amide hydrogen will form an H-bond (Figure 3).

**Figure 3:**
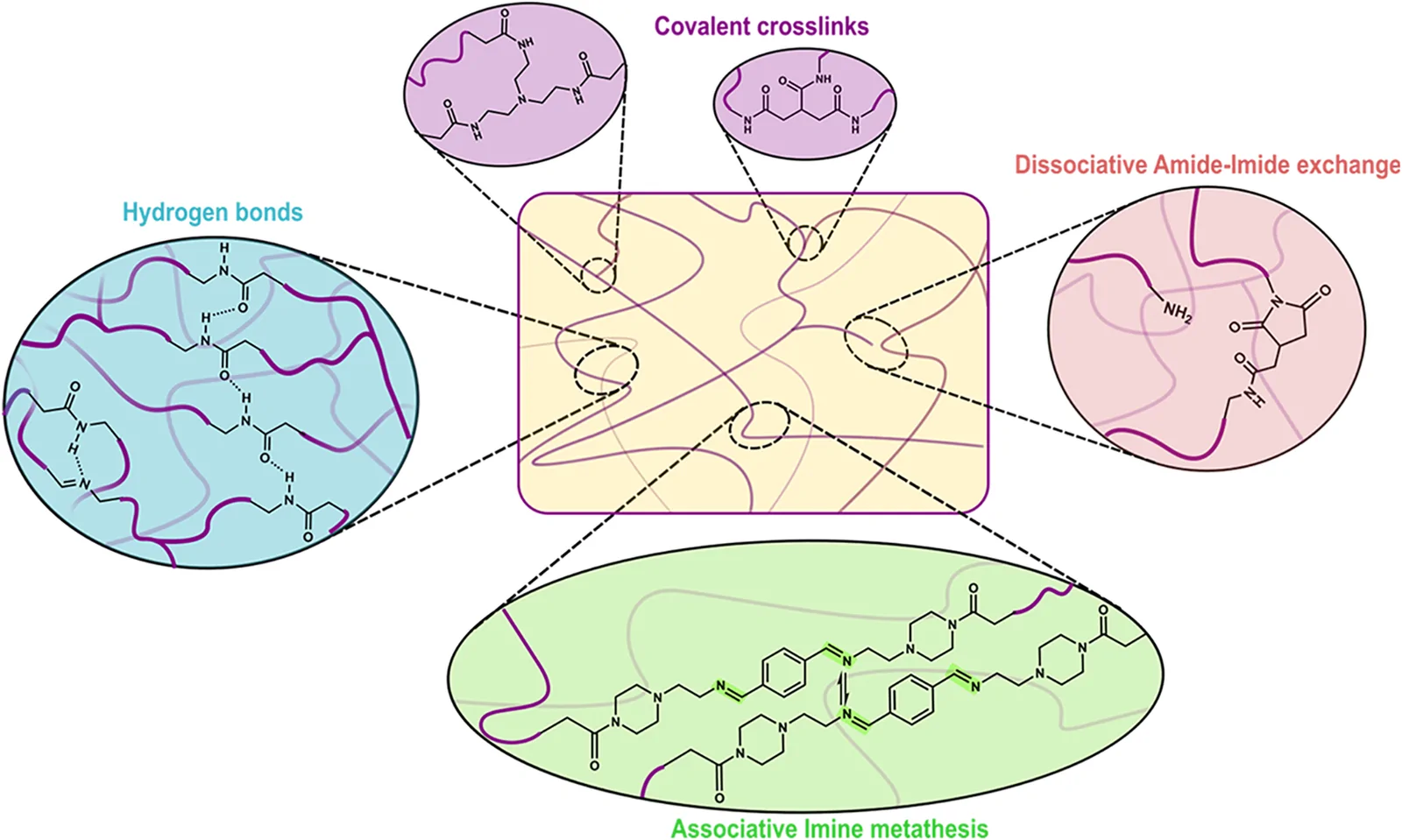
Schematic representation depicting the main covalent and physical cross-links and the dynamic moieties of a typical PAI network.

Finally, PAI-D230–0.75 exhibits a slightly higher *E*′_Rubbery_ than PAI-D230–0.25 due to the further reduction in the D230 proportion, but less significant than that observed in PAI-D230–0.50. Indeed, in this case, the presence of secondary amides is considerably lower than that of tertiary amides, resulting in less pronounced H-bonding phenomena.

The α transition was found to be affected by [PPO], shifting to higher temperatures when the D230 proportion is decreased. The evolution of tan δ with temperature also reveals the effect of the IDA monomer on the network architecture. Increasing the IDA content leads to a progressively more inhomogeneous network, as demonstrated by an increasingly pronounced shoulder peak appearing along the PAI-D230 series. This is ascribable to the high rigidity of the IDA structure, which is conferred by the presence of two imine groups adjacent to an aromatic ring. The aromatic character of the monomer could also cause segregation phenomena during the curing process, resulting in the formation of some rigid domains. *T*_*g*_ *s* determined by DSC (Figure S5) confirm this hypothesis. Indeed, as shown in Table 2, *T*_*g*_ *s* exhibits relatively small variations with increasing IDA content, while the tan δ peak shifts significantly to higher temperatures, resulting in progressively more inhomogeneous. This could confirm the presence of different domains in which the α transition occurs at slightly different temperatures.

### 3.4 Dynamic Properties

Temperature-dependent stress relaxation experiments were performed by DMA at temperatures ranging from 110 to 190 °C. The relaxation moduli *E*′*(t)* were monitored over durations ranging from 10 to 10,000 s. For all of the tested materials, a progressively smaller modulus was observed at the beginning of the experiment *(E*′*(t = 0))* when the temperature was increased, indicating that the cross-link density decreased, as typically observed for a dissociative mechanism (Figure S6).^26^ Considering the presence of multiple dynamic bonds in the network and the heterogeneity of the materials’ microstructure, the direct application of the typical Kohlrausch–Williams–Watts^27,28^ stretched exponential decay model could be ambiguous. As shown in Figure 3, three possible reactions may be involved in the network rearrangement during stress relaxation. While imine metathesis and amide exchange occur independently, the release of primary amines during the amide dissociation can promote the imine transimination. To investigate more deeply the relaxation modes involved in the overall stress dissipation process, an analysis of the continuous relaxation time spectrum (CRS) was performed using the open-source software package “PyReSpect.”^29^ As evidenced in Figure 4, the CRS model was found to accurately capture the experimental stress relaxation data.

**Figure 4:**
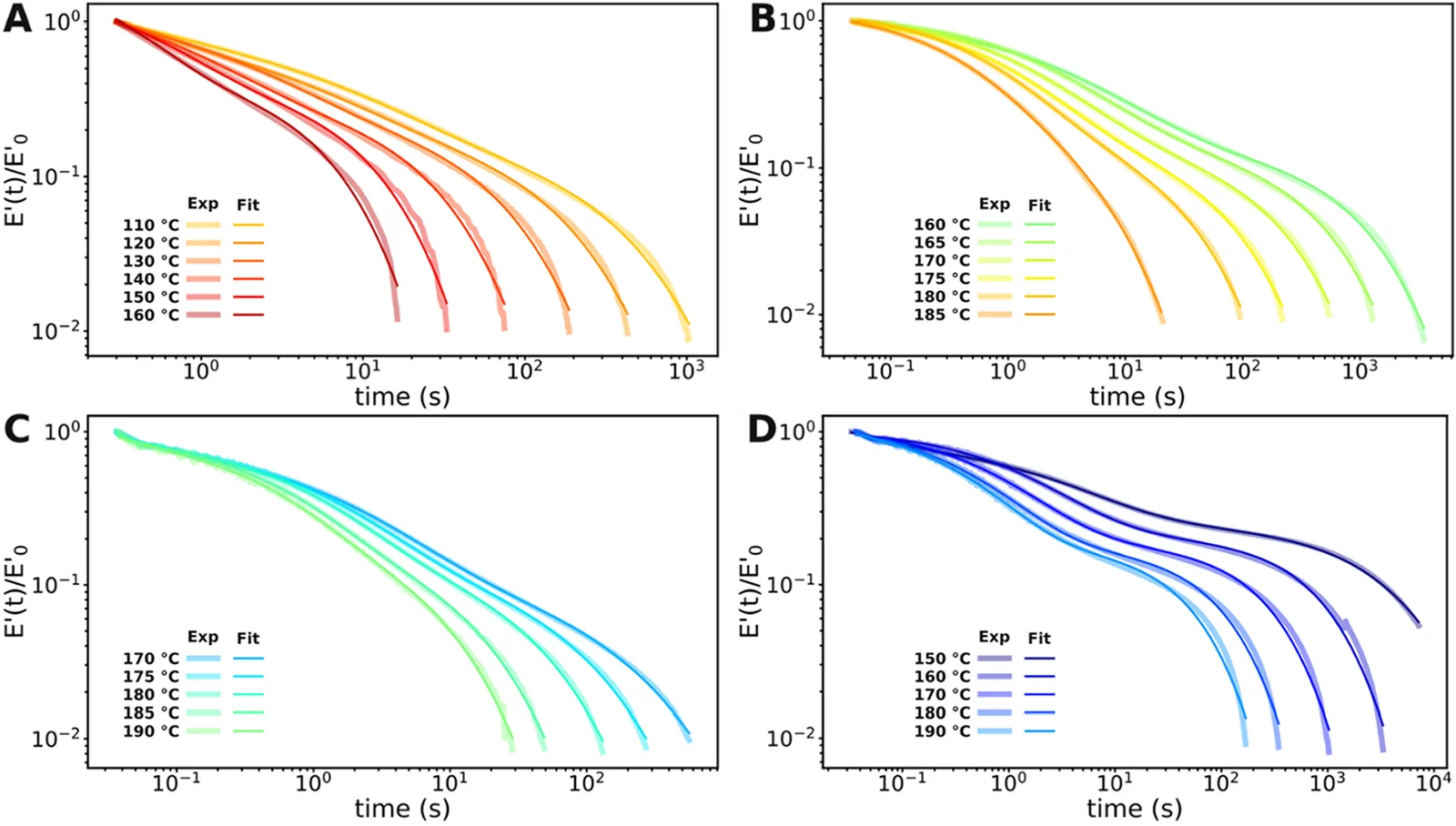
Stress relaxation results and corresponding CRS fitted curves for: (A) PAI-D230–0.25; (B) PAI-D230–0.50; (C) PAI-D230–0.75; and (D) PAI-HMDA-0.25.

The calculation of the CRS allowed decomposition of the complex rheological response of the prepared networks into discrete relaxation modes, as illustrated in Figure 5A for PAI-D230–0.50 at 170 °C. Figure 5(D–F) shows the CRS spectra, referred to as *h*(τ), for each tested temperature of the PAI materials. As observed in all of the spectra, the relaxation processes are generally dominated by two main stress dissipation modes that occur in different time regimes. Increasing the temperature causes both relaxation modes to shift toward shorter relaxation times, demonstrating different temperature dependencies, which in some cases result in partial peak overlap. The observation of only two dominant stress dissipation modes suggests that imine metathesis and imine transimination occur over similar time scales and therefore contribute to the same relaxation process. As a first approximation, the first relaxation peak was attributed to exchange reactions involving imine groups, while the long-range relaxation mode was ascribed to the generally slower amide exchange.^20–22^ Indeed, as previously discussed, the decrease in cross-link density, evidenced by the progressively lower values of *E*′*(t = 0)*, implies the formation of cyclic imide already during the previous thermal equilibration phase. Hence, primary amines released in this context add to those already formed during the curing process, contributing to relaxation modes from the very beginning of the stress relaxation process.

**Figure 5:**
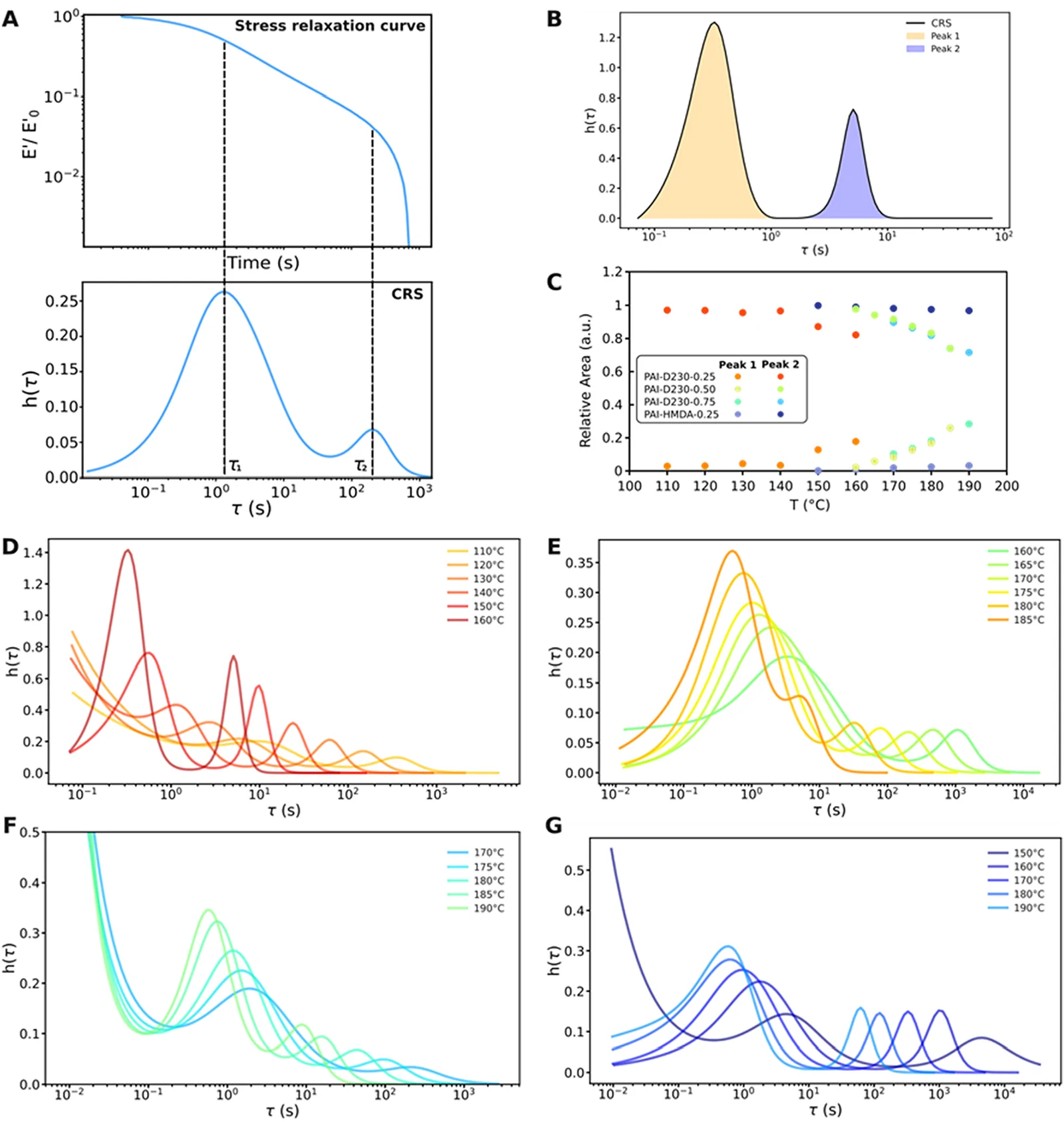
(A) Stress relaxation and corresponding CRS spectra of PAI-D230–0.50 at 170 °C; (B) graphical representation of the relaxation peak integration in the CRS spectra of PAI-D230–0.25 at 160 °C; and (C) evolution with temperature of the relative areas of the relaxation peaks. CRS spectra of the materials: (D) PAI-D230–0.25; (E) PAI-D230–0.50; (F) PAI-D230–0.75; and (G) PAI-HMDA-0.25.

To investigate the contribution of each relaxation mode to the overall process, the area under each peak was integrated (Figure 5B), and the results were normalized to the total area subtended by the CRS (Figures S7–S10). As shown in Figure 5C, the relative area of the long-range relaxation modes decreases with increasing temperature, while the contribution of the short-range mode generally increases. Higher temperatures promote amide dissociation, and the resulting release of higher concentrations of primary amines boosts the transimination reaction, thereby increasing the contribution of the short-range mode to the overall relaxation process. Increasing the IDA content from 25 to 50% accentuates this behavior, increasing the imine concentration in the network and promoting both metathesis and transimination. No significant differences were observed upon further increasing the IDA content to 75%, indicating that the concentration of secondary amide groups in the network is too low to further influence transimination exchange. Additionally, a rapid relaxation process is observed below 0.1 s for the PAI-D230–0.75 material (Figure 5F), likely due to the breaking of hydrogen bonds caused by the mechanical deformation of the network. Interestingly, when the polyether amine spacer D230 was replaced by HMDA, the relative contributions of the two relaxation modes remained almost unaltered across the temperature range, suggesting that the network architecture exerts a controlling effect on the relaxation mechanism, thereby limiting the transimination exchange process (Figure 5C).

To corroborate the inferences drawn from the CRS analysis, temperature-dependent FTIR spectroscopy was conducted on the PAI-D230 series materials to study the dissociation equilibria of the amide groups over a temperature range from 100 to 200 °C (Figures S14–S16). The FTIR spectra were deconvoluted in the 1900–900 cm^–1^ region, monitoring the intensities of the amide (1646 cm^–1^) and imide (1696 cm^–1^) bands, as shown in Figure 6A. The ratio of the normalized intensities of the amide and imide bands decreases with increasing temperature, reflecting the reduction in amide concentration due to the imide ring formation (Figure 6B). The similar trends observed for all of the prepared PAI-D230 materials suggest that the impact of IDA content on the network architecture does not affect the extent of amide dissociation, although it could significantly influence the activation energy of the exchange process, as will be discussed further.

**Figure 6:**
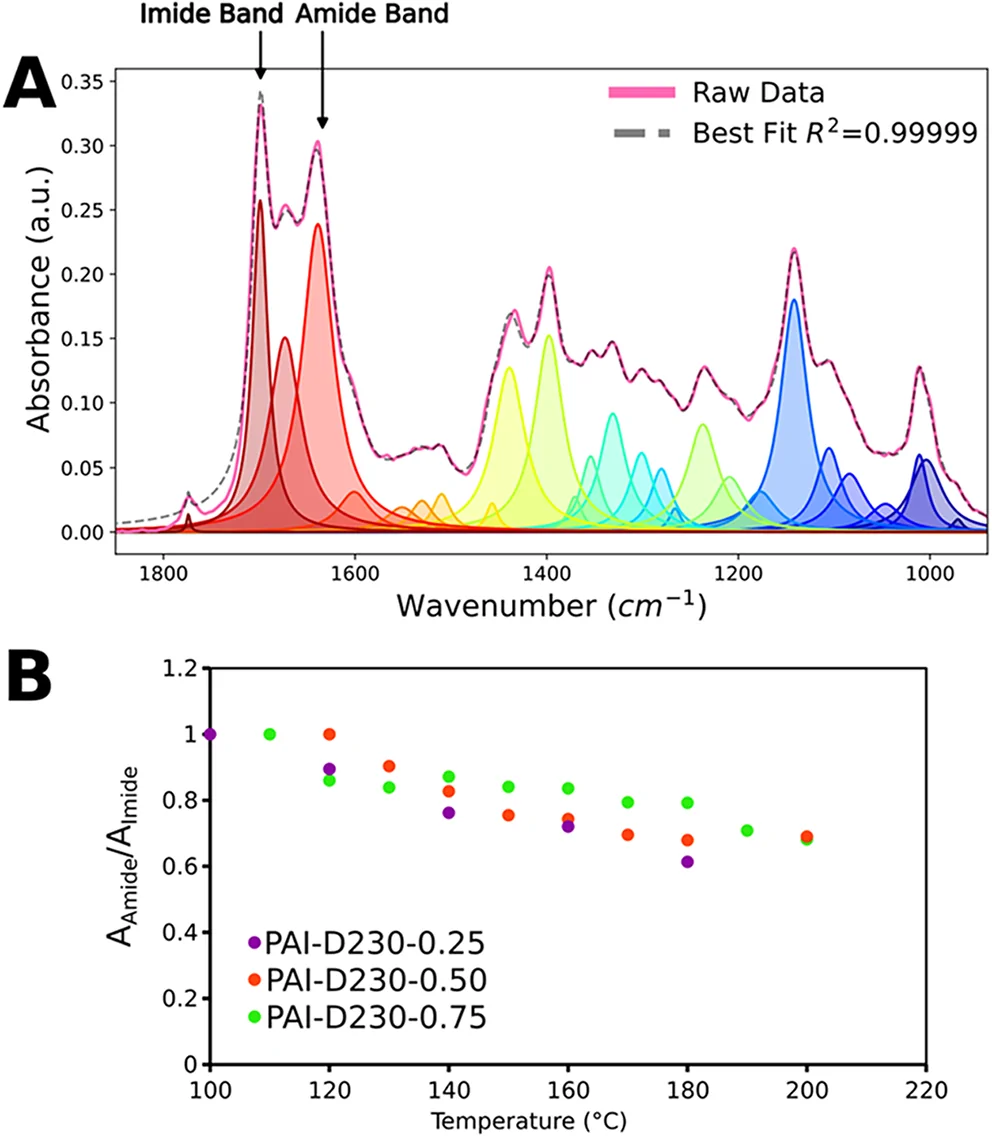
(A) Deconvoluted bands of the FTIR spectrum of PAI-D230–0.75 at 170 °C and best fitted curve (dashed black) compared to the original spectrum (pink); (B) ratio of the normalized intensities of the diamide and imide bands as a function of temperature for the PAI-D230 series.

The relaxation times τ_1_^CRS^ and τ_2_^CRS^ assigned to the maxima of the *h*(τ) peak (Table S1) were used to construct the CRS Arrhenius plots (Figure S11A–D) and to calculate the activation energies, *E*_*a*_^CRS^, of the two relaxation modes (Table 3).

**Table 3:** Kinetic Activation Energies of the Stress Relaxation of the Network Calculated from the Double Element Kohlrausch–Williams–Watts (DKWW) Fittings and CRS *h*(τ) Maxima

sample	*E*_*a*,1_^CRS^ (kJ/mol)	*R* ^2^	*E*_*a*,2_^CRS^ (kJ/mol)	*R* ^2^	*E*_*a*,1_^DKWW^ (kJ/mol)	*R* ^2^	*E*_*a*,2_^DKWW^ (kJ/mol)	*R* ^2^
PAI-D230–0.25	97 ± 4	0.993	120 ± 2	0.999	63 ± 3	0.992	111 ± 2	0.998
PAI-D230–0.50	118 ± 4	0.994	313 ± 33	0.991	130 ± 5	0.995	334 ± 15	0.992
PAI-D230–0.75	101 ± 7	0.989	275 ± 12	0.994	96 ± 8	0.978	235 ± 12	0.993
PAI-HMDA-0.25	87 ± 7	0.982	155 ± 9	0.993	91 ± 6	0.988	212 ± 13	0.989

Short-range relaxations exhibited less pronounced temperature-dependent times with *E*_*a*,1_^CRS^ ranging from 87 to 118 kJ/mol. In contrast, long-range modes were characterized by considerably higher *E*_*a*,2_^CRS^ values. Raising the proportion of IDA typically resulted in higher activation energies for both relaxation modes, with a particularly pronounced effect on the amide exchange process. When analyzing temperature-dependent rheological data, it must be considered that the activation energy reflects not only the bond exchange but also contributions from polymer segment dynamics and network reconfiguration.^27,30^ Therefore, the temperature dependence of each relaxation mode is driven by the dominant among multiple contributing factors. Raising the proportion of IDA typically results in slightly higher activation energies for the imine-related relaxation mode, with a particularly pronounced effect on the amide exchange process. Indeed, increasing the IDA content leads to a lower concentration of secondary amide groups in the network, which are prone to imide ring formation. This slows down the bond exchange process and makes it the rate-determining step of the relaxation mode toward network rearrangement, thereby increasing the temperature dependence of the process. In PAI-D230–0.50, the lower concentration of secondary amide is associated with a highly cross-linked matrix. As previously discussed, this arises from both covalent and physical cross-linking, which hampers network reconfiguration. In this case, the low reaction rate and the highly hindered network compete to control the relaxation process, resulting in an extremely high *E*_*a*,2_^CRS^ value. Replacing the polyether spacer D230 with the shorter, purely aliphatic HMDA increases the cross-link density, creating a highly constrained network. This maintains the polymer chains closer together, facilitating associative mechanisms such as imine metathesis and transimination, slightly lowering *E*_*a*,1_^CRS^.

From the other side, the shorter chain length of the spacer determines a higher concentration of secondary amides than in PAI-D230–0.25, which results in a more pronounced dissociation process, as demonstrated by higher *E*_*a*,2_^CRS^. The higher relaxation times observed for PAI-HMDA-0.25 point out that in this contest, the network rearrangement, constrained by a highly cross-linked matrix, becomes the dominant factor for the stress relaxation process.

Finally, to confirm the results of the CRS analysis, a double Kohlrausch–Williams–Watts (DKWW) model, based on a linear combination of two stretched exponential decay functions (eq 2), was employed to describe the stress relaxation process

2 $$\frac{E^{\prime }\left(t\right)}{E_{o}^{\prime }}=Ae^{{\left(\frac{t}{\tau_{1}}\right)}^{\beta_{1}}}+\left(1-A\right)e^{{\left(\frac{t}{\tau_{2}}\right)}^{\beta_{2}}}$$

where $\frac{E^{\prime }\left(t\right)}{E_{o}^{\prime }}$ is the relaxation modulus normalized to the its value at the beginning of the stress relaxation process; τ_1_ and τ_2_ are the characteristic relaxation times of the relaxation path ascribable to the two dynamic bonds; β_1_ and β_2_ are the stretching parameters that quantitatively describe the broadness of the distribution of relaxation modes for each relaxation path; and *A* is the pre-exponential factor. The characteristic relaxation times and stretching parameters were extracted by fitting the normalized relaxation modulus at each temperature to eq 2, as shown in Figure S12. They were then employed in the calculation of the average relaxation times ⟨τ⟩ ^28,31^

3 $$\left\langle \tau \right\rangle =\frac{\tau }{\beta }\Gamma \left(\frac{1}{\beta }\right)$$

The DKKW model was found to properly fit the experimental stress relaxation data. As revealed by the CRS analysis, the faster relaxation mode was attributed to imine-related exchange reactions, while amide exchange was considered responsible for the long-range relaxation mode. The Arrhenius plot built from the calculated ⟨τ⟩ values (Figure S13 and Table S2) was used to calculate the activation energies for the two relaxation modes, *E*_*a*,1_^DKWW^ and *E*_*a*,2_^DKWW^ (Table 3), thus confirming the results obtained from the CRS analysis.

### 3.5 Creep Experiments

Creep experiments were conducted on the materials of the PAI-D230 series in the temperature range between 110 and 190 °C (Figure S17). As shown in Figure 7, where the natural logarithm of the viscosity is plotted against the inverse of the temperature, the three studied materials exhibit different evolutions of viscosity with temperature.

**Figure 7:**
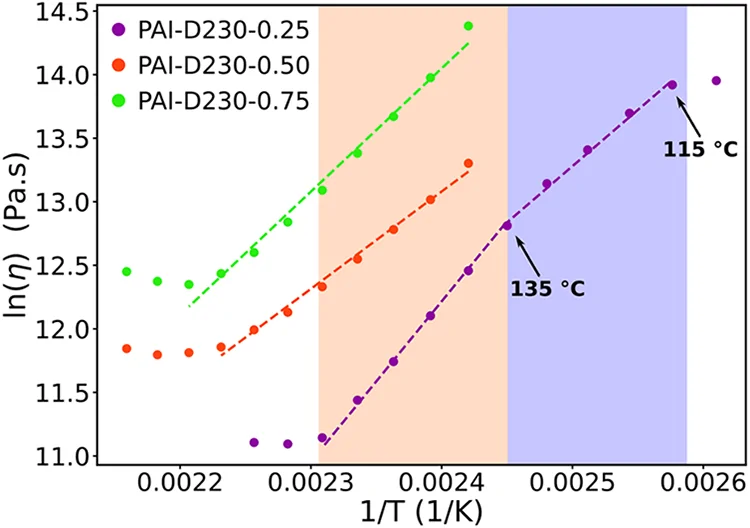
Arrhenius plot obtained from creep measurements of PAI-D230 materials.

Consistent with its lower proportion of IDA, PAI-D230–0.25 behaves as a typical dissociative system, displaying different viscoelastic flow regimes as the temperature increases.^32^ At low temperatures, viscous flow is almost exclusively controlled by exchange reactions occurring under conditions of nearly constant cross-link density. As the temperature increases to 115 °C, dissociation becomes significant, altering the network structure. However, bond exchange still dominates the flow process. Within this temperature range, viscosity follows an Arrhenius dependence characterized by an activation energy (*E*_*a*,1_^η^) of 73 kJ/mol (Table 4). Above 135 °C, the viscosity decreases more sharply (*E*_*a*,2_^η^ = 99 kJ/mol), due to a progressive reduction in the cross-link density of the network. Due to the higher *T*_tanδ_, both PAI-D230–0.50 and PAI-D230–0.75 were studied at higher temperatures, with only one viscous regime observed within the investigated range. PAI-D230–0.50, in particular, exhibits very low temperature dependence (*E*_*a*_^η^ = 64 kJ/mol), probably due to its higher average cross-link density. Furthermore, the activation energy of viscous flow was found to be lower in PAI-D230–0.75 (*E*_*a*_^η^ = 81 kJ/mol) than in PAI-D230–0.25­(*E*_*a*,2_^η^ = 99 kJ/mol), *i.e*., the activation energy of flow within the same temperature range. This is consistent with the higher proportion of IDA, which reduces the dissociative character of the dynamic network.

**Table 4:** Activation Energies of Viscous Flow Calculated by Linearly Fitting the Viscosities as a Function of 1/*T*

sample	*E*_*a*,1_^*η*^ (kJ/mol)	*R* ^2^	*E*_*a*,2_^*η*^ (kJ/mol)	*R* ^2^
PAI-D230–0.25	73 ± 3	0.994	99 ± 1	0.999
PAI-D230–0.50	64 ± 3	0.990		
PAI-D230–0.75	81 ± 4	0.989		

### 3.6 Adhesive Properties

The potential use of the PAI-D230 materials as adhesives was investigated by means of lap shear tests. Since the formation of the network structure for this kind of material involves the release of MeOH, the typical approach in which the monomer mixture reacts directly between the substrate surfaces is not feasible. The PAI materials were prepared using the usual procedure and pressed to form 0.2 mm thickness films. These films were then cut into rectangular specimens with dimensions in accordance with the UNE standard, positioned between the two substrate surfaces (previously treated as described in Section 2.5), clamped, and finally heated at 160 °C in a conventional oven. Lap shear tests were conducted following the UNE-EN ISO 1465:2009 standard test method.^23^

The adhesion strength of the PAI materials decreases as the proportion of IDA increases, showing lap shear strength values ranging from 2.6 MPa for PAI-D230–0.75 to 8.4 MPa for PAI-D230–0.25 (Table 5). The proportion of IDA in the formulation has a great impact on the viscous flow of the materials, as demonstrated by creep experiments conducted at high temperatures (Section 3.4). A higher content of the IDA monomer increases the viscosity, which hampers adhesion to the substrate surface. Moreover, higher proportions of IDA result in a lower content of secondary amide groups in the network, reducing the formation of hydrogen bonds between the adhesive and the substrate surface. Anyway, all of the materials exhibit adhesive-type failure (Figure S19), which could probably be caused by the extremely high viscosity at 160 °C of all of the samples, as observed in the creep experiments.

**Table 5:** Data from Lap Shear Tests of the PAI-D230 Materials

sample	*F*_max_ ^a^ (N)	lap shear strength^b^ (MPa)
PAI-D230–0.25	2641 ± 125	8.4 ± 0.4
PAI-D230–0.50	1377 ± 74	4.4 ± 0.2
PAI-D230–0.75	797 ± 34	2.6 ± 0.1

a Force at break was obtained in lap shear tests.

b Stress at break was calculated from the force–displacement curves.

In order to investigate whether the dynamic character of the PAI adhesives could confer debonding ability, lap shear tests at 160 °C were performed. The very low viscosity of all of the tested adhesives made it impossible to measure the shear stress under standard conditions, as debonding already occurred during the thermal equilibration. A different procedure was then set up in a conventional oven, connecting a weight of 60 g (0.59 N) to the lower aluminum bar after 10 min of sample equilibration at 160 °C. In all cases, a total debonding was observed in less than 20 min for all of the PAI adhesives. Although this setup cannot be used for comparative purposes, it provides a qualitative demonstration of the debonding ability of these materials under extremely low loads. Figure 8 shows the PAI adhesives after debonding.

**Figure 8:**
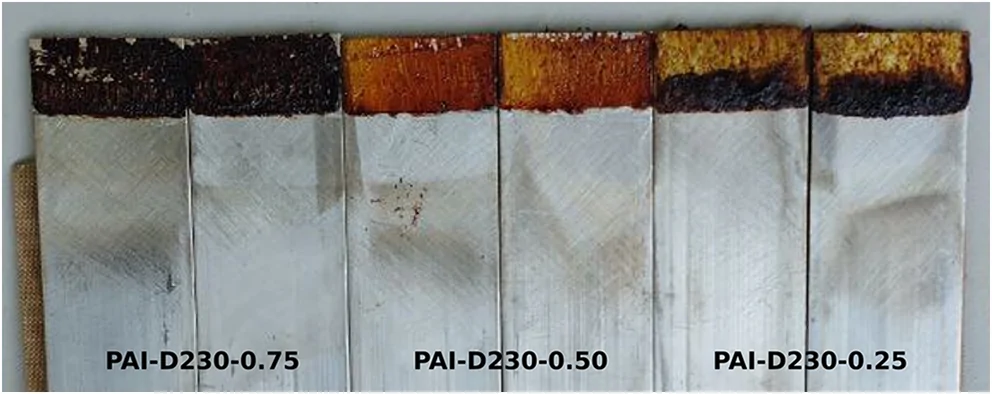
PAI adhesives after debonding at 160 °C, applying a 0.59 N constant force.

## 4 Conclusions

Dynamic covalent networks were prepared through an amidation reaction involving methyl tricarballylate (MT) and commercially available bifunctional (Jeffamine D230) and trifunctional (TREN) amines as well as an imine-containing secondary diamine (IDA) synthesized by us. The resulting networks contain secondary amide groups, which can undergo dissociative transamidation reactions mediated by the neighboring groups and imines capable of associative metathesis reactions. Although imine metathesis and amide exchange occur independently, the release of primary amines during imide dissociation was found to promote the imine transimination.

Regarding the dynamic behavior, the presence of the two dynamic groups in the network generally boosted the stress relaxation process. Increasing the proportion of IDA monomer not only led to a higher content of imine groups but was also found to drastically affect the network architecture, greatly impacting the activation energies of the two relaxation modes. Although the overall stress relaxation process was dominated by the slower amide exchange relaxation mode, increasing the proportion of IDA led to a higher contribution of the imine-related relaxation processes, as revealed by the analysis of the continuous relaxation spectra.

Creep experiments at high temperatures show how the IDA content can be used to adjust the flow behavior of PAI materials, shifting from a purely dissociative viscous profile obtained at low IDA proportions to a mainly associative behavior at higher IDA content. Moreover, the viscous flow properties extrapolated from creep experiments corroborate what was observed during adhesion tests. The PAI materials were tested using lap shear measurements, showing a lap shear strength of up to 8.4 MPa at room temperature when the proportion of IDA does not exceed 25%. Exposing the formulations to temperatures around 160 °C resulted in a severe drop in adhesive strength, making the PAI adhesives easily debondable even under extremely low load (0.59 N).

This work demonstrated the potential of amide exchange and imine metathesis as a dual dynamic system. This suggests that developing these poly­(amide-imine) materials could pave the way for new advancements in covalent adaptable networks. This would unlock rheological properties that are not possible with traditional thermosets, thus expanding their potential applications in high-performance fields.

## Supplementary Material



## Notes

Description of monomer synthesis and characterization; thermogravimetric, thermomechanical, and thermal characterization, stress relaxation experiments (including continuous relaxation spectra and KWW model analysis), high-temperature FTIR spectroscopy, creep experiments, and lap shear testing of the poly­(amide-imine) dynamic networks (PDF)
