# Supplementary material for: Reprocessable Covalent Adaptable Networks Enabled by Transamidation and Imine Metathesis Exchanges
Source: ACS Polym Au. 2026 Aug 31;6(5):1488–98. doi: 10.1021/acspolymersau.6c00150 (PMC13673875; doi:10.1021/acspolymersau.6c00150)
Supplement: Supplementary file 1 [file lg6c00150_si_001.pdf]

Supporting Information

Reprocessable Covalent Adaptable Networks Enabled by Transamidation and Imine Metathesis Exchanges

Tommaso Telatin[1], Thijs Van Casteren[1], Silvia De la Flor[2], Àngels Serra[1], Xavier Montané[1,*]

[1] Universitat Rovira i Virgili, Department of Analytical and Organic Chemistry, C/ Marcel·lí Domingo 1, 43007 Tarragona, Spain

[2] Universitat Rovira i Virgili, Department of Mechanical Engineering, Av. Països Catalans 26, 43007 Tarragona, Spain

[*] Corresponding author.

E-mail address: xavier.montane@urv.cat (X.M.)

Table of contents:

1. Monomer synthesis **S3**

1.1. Synthesis of methyl tricarballylate (MT) **S3**

1.2. Synthesis of the imine-containing secondary diamine monomer (IDA) **S4**

2. Thermogravimetric analysis **S6**

3. Thermomechanical characterization by DMA **S7**

4. Thermal characterization by DSC **S8**

5. Stress relaxation experiments **S9**

5.1. Continuous relaxation spectra **S10**

5.2. KWW model **S16**

6. High temperature FTIR spectroscopy **S19**

7. Creep experiments **S22**

8. Lap shear tests **S23**

References **S24**

1. Monomer synthesis

1.1. Synthesis of methyl tricarballylate (MT)

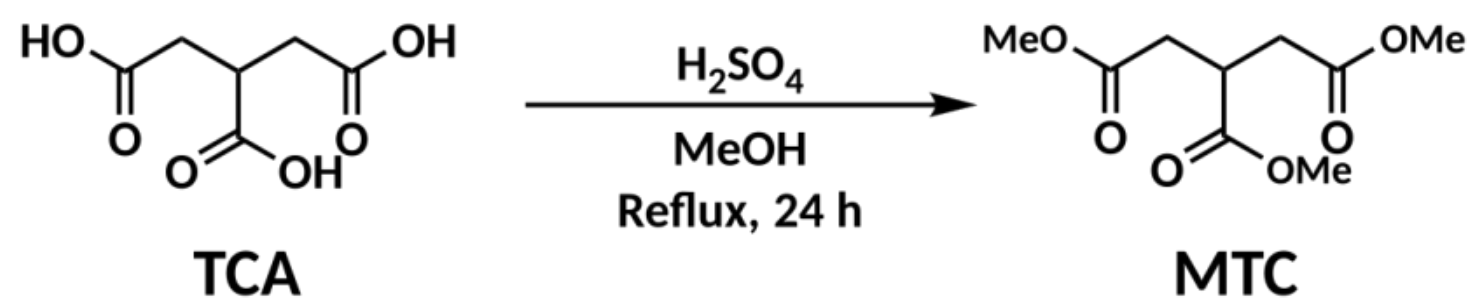

Scheme S1. Synthesis of methyl tricarballylate (MT) from tricarballylic acid (TCA).

Methyl tricarballylate (MT) was synthesized according to a procedure previously described in the literature.[1]

Tricarballylic acid (TCA) (25.00 g, 142.0 mmol) and 100 mL of MeOH (1.42 mol) were added to a 250 mL flask equipped with a magnetic stirrer and a reflux condenser. Subsequently, 1 mL of H_2SO_4 was slowly added to the mixture, and the temperature was raised to reflux and stirred for 24 h. The solution was then cooled to room temperature, poured into ice water (100 mL), and stirred for 30 min. The product was extracted with Et_2O (5 x 100 mL), and the organic phase was dried over anhydrous $MgSO_4$, filtered and concentrated in a rotary evaporator, yielding MT as colorless transparent liquid (99%).

Methyl tricarballylate (MT): ^{1}H NMR ($CDCl_3$; δ, ppm): 3.64 (s, 3H), 3.62 (s, 6H), 3.20 (m, 1H) and 2.63 (m, 4H) (**Figure S1**).

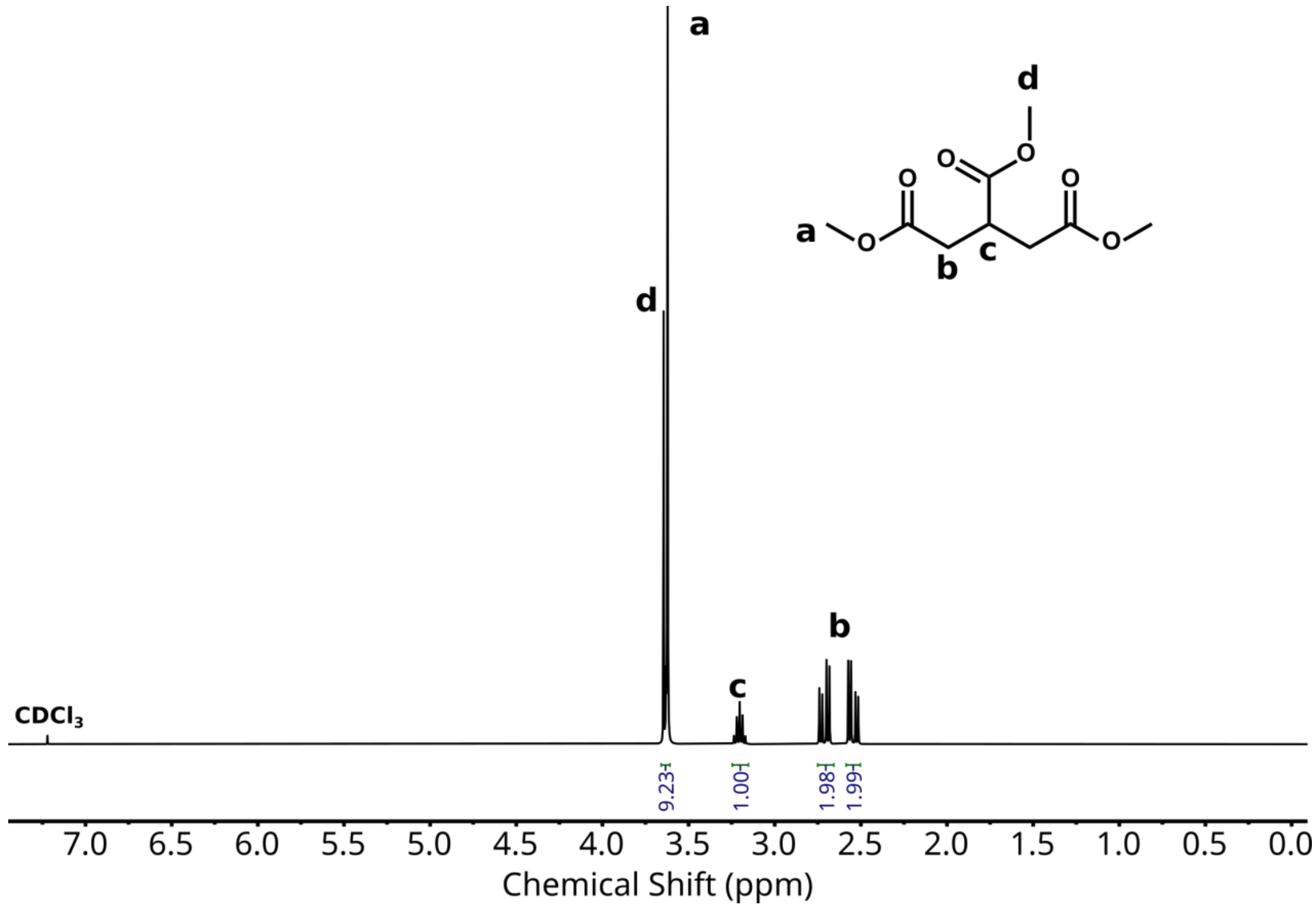

Figure S1. ^{1}H NMR spectrum of methyl tricarballylate (MT) in $CDCl_3$.

1.2. Synthesis of the imine-containing secondary diamine monomer (IDA)

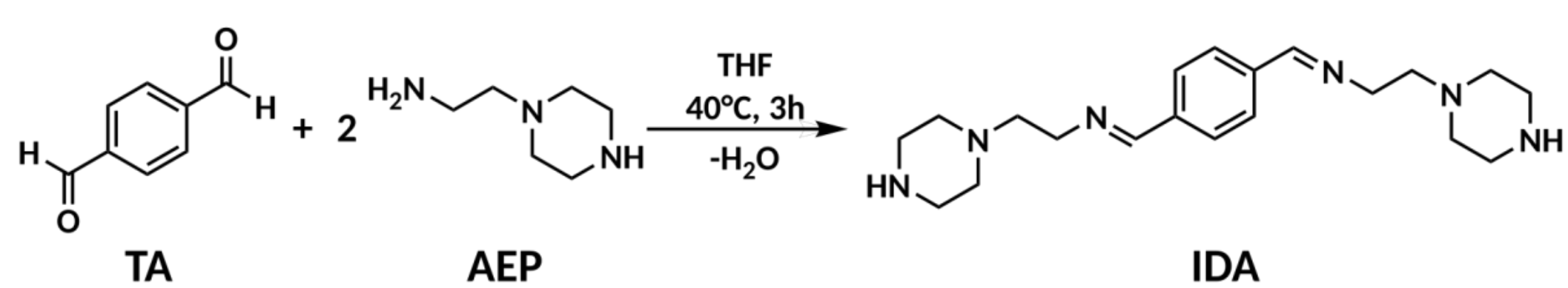

Scheme S2. Synthesis of the imine-containing secondary diamine monomer (IDA) from terephthalaldehyde (TA) and 2-aminoethyl piperazine (AEP).

Terephthalaldehyde (TA) (3.42 g, 25.5 mmol) was dissolved in 80 mL of THF in a round bottom flask equipped with a magnetic stirrer and a reflux condenser. 2-aminoethyl piperazine (AEP, 6.60 g, 51.0 mmol) was added dropwise to the solution under stirring, and the temperature was raised

to reflux for 8 h. The solution was then dried over anhydrous $MgSO_4$, filtered and concentrated in a rotary evaporator, yielding AEP as an orange solid. ^1H NMR ($CDCl_3$; δ, ppm): 8.26 (s, 2H), 7.71 (m, 4H), 3.74 (t, 4H), 2.85 (t, 8H), 2.65 (t, 4 H), 2.48 (t, 8 H) (**Figure S2**).[2]

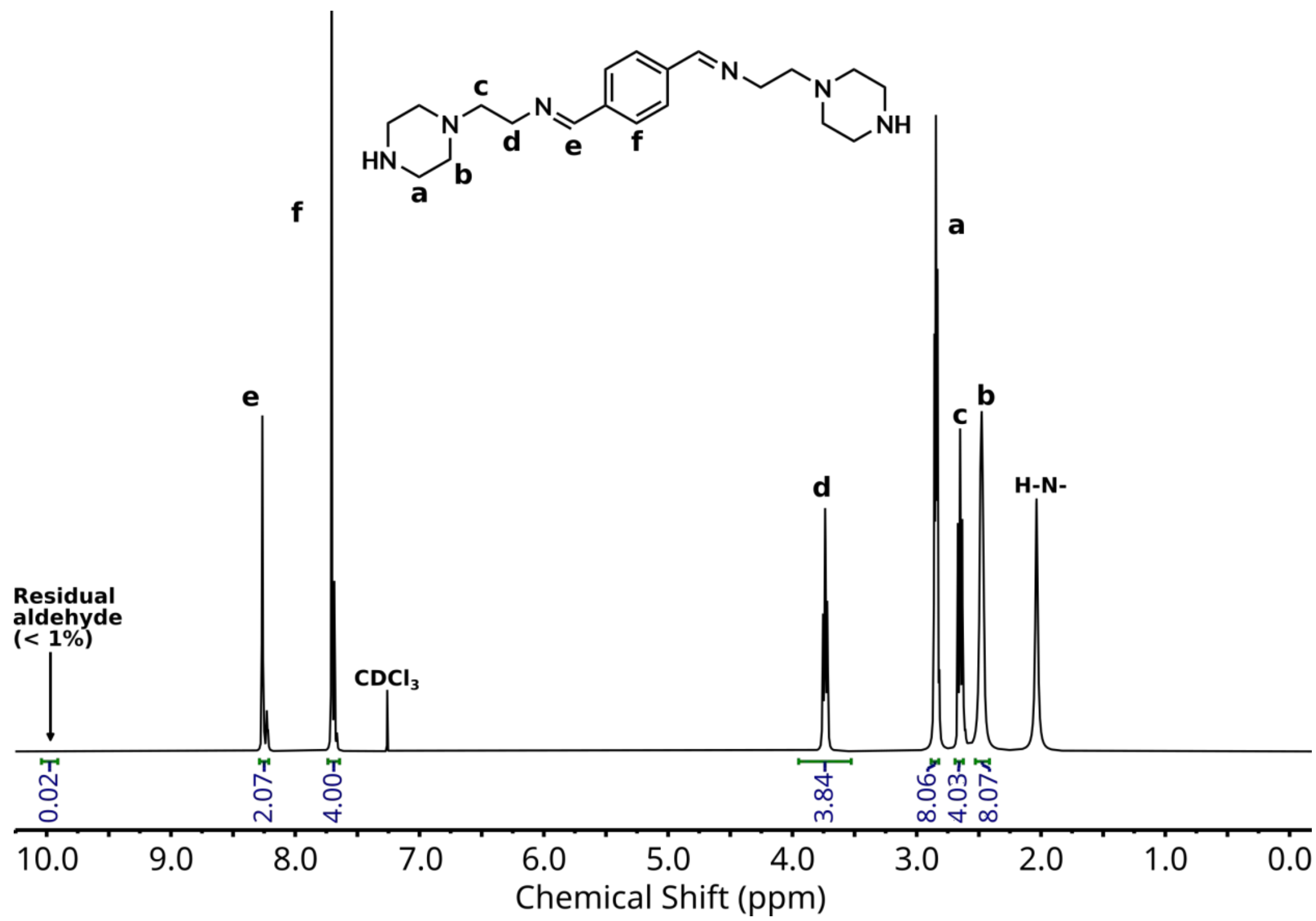

Figure S2. ^1H NMR spectrum of the imine-containing secondary diamine monomer (IDA) in $CDCl_3$.

2. Thermogravimetric analysis

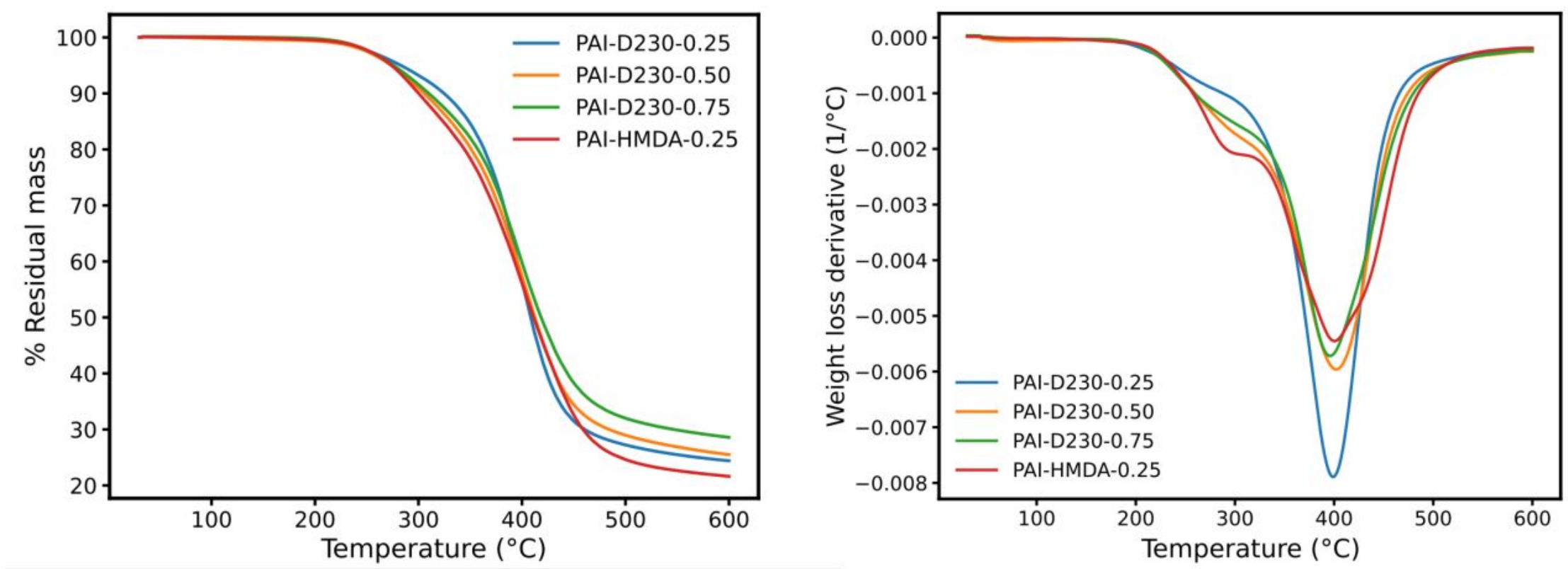

Figure S3. TGA and DTGA curves for the PAI materials in N_2 atmosphere.

3. Thermomechanical characterization by DMA

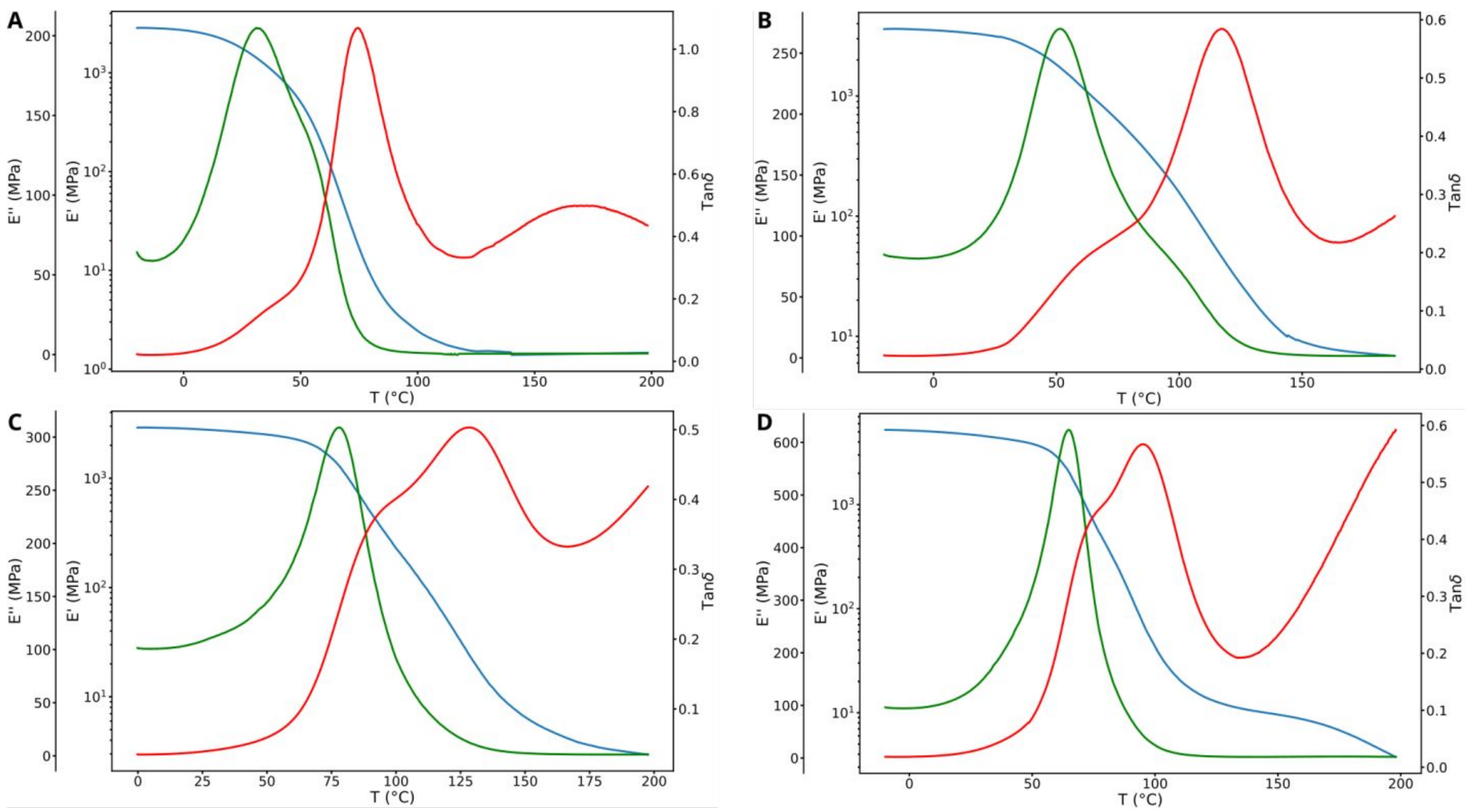

Figure S4. Evolution of storage modulus (blue line), loss modulus (green line), and tan δ (red line) with temperature for the materials: A) PAI-D230-0.25; B) PAI-D230-0.50; C) PAI-D230-0.75 and D) PAI-HMDA-0.25.

4. Thermal characterization by DSC

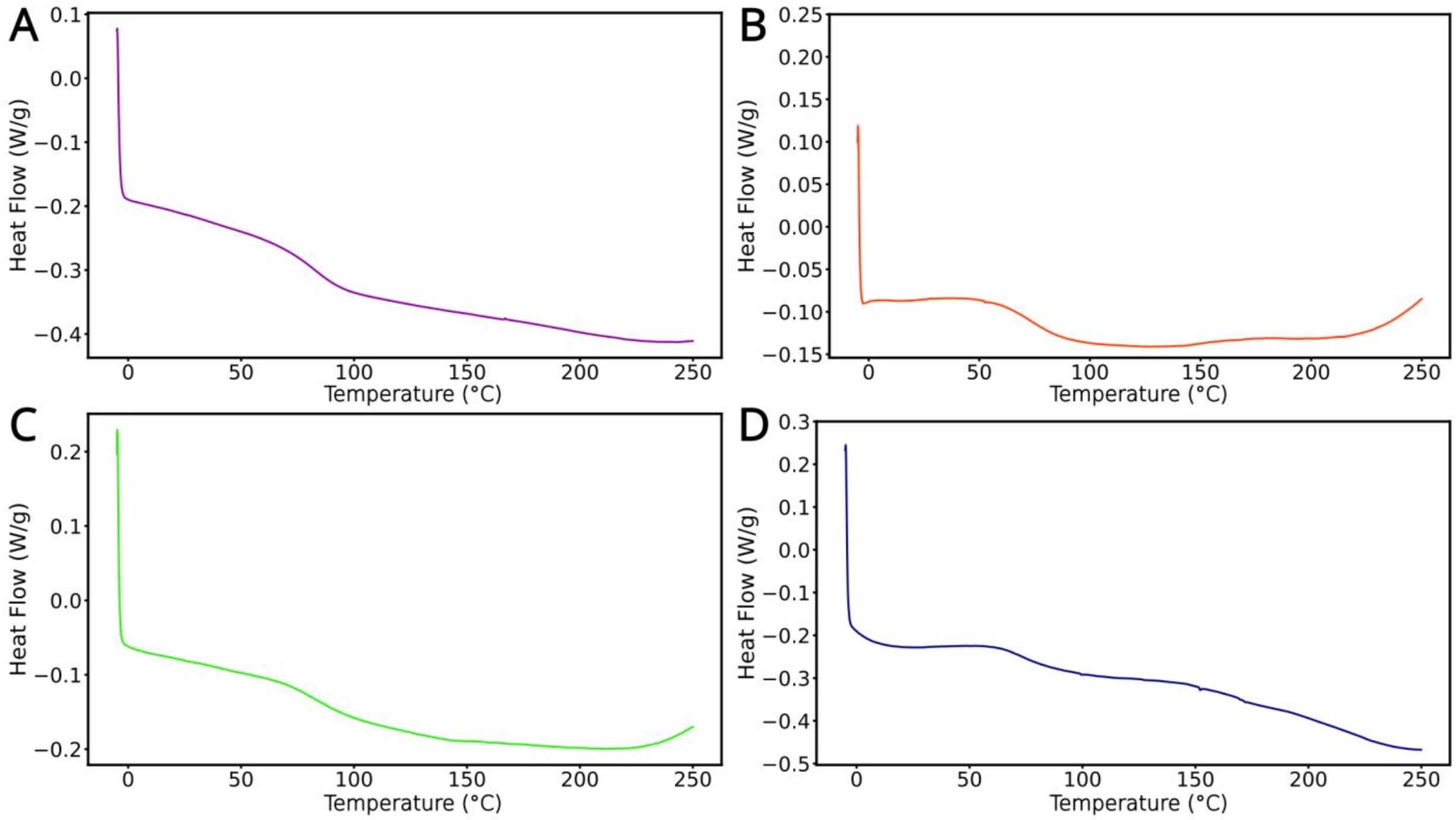

Figure S5. DSC thermograms of the materials: A) PAI-D230-0.25; B) PAI-D230-0.50; C) PAI-D230-0.75 and D) PAI-HMDA-0.25.

5. Stress relaxation experiments

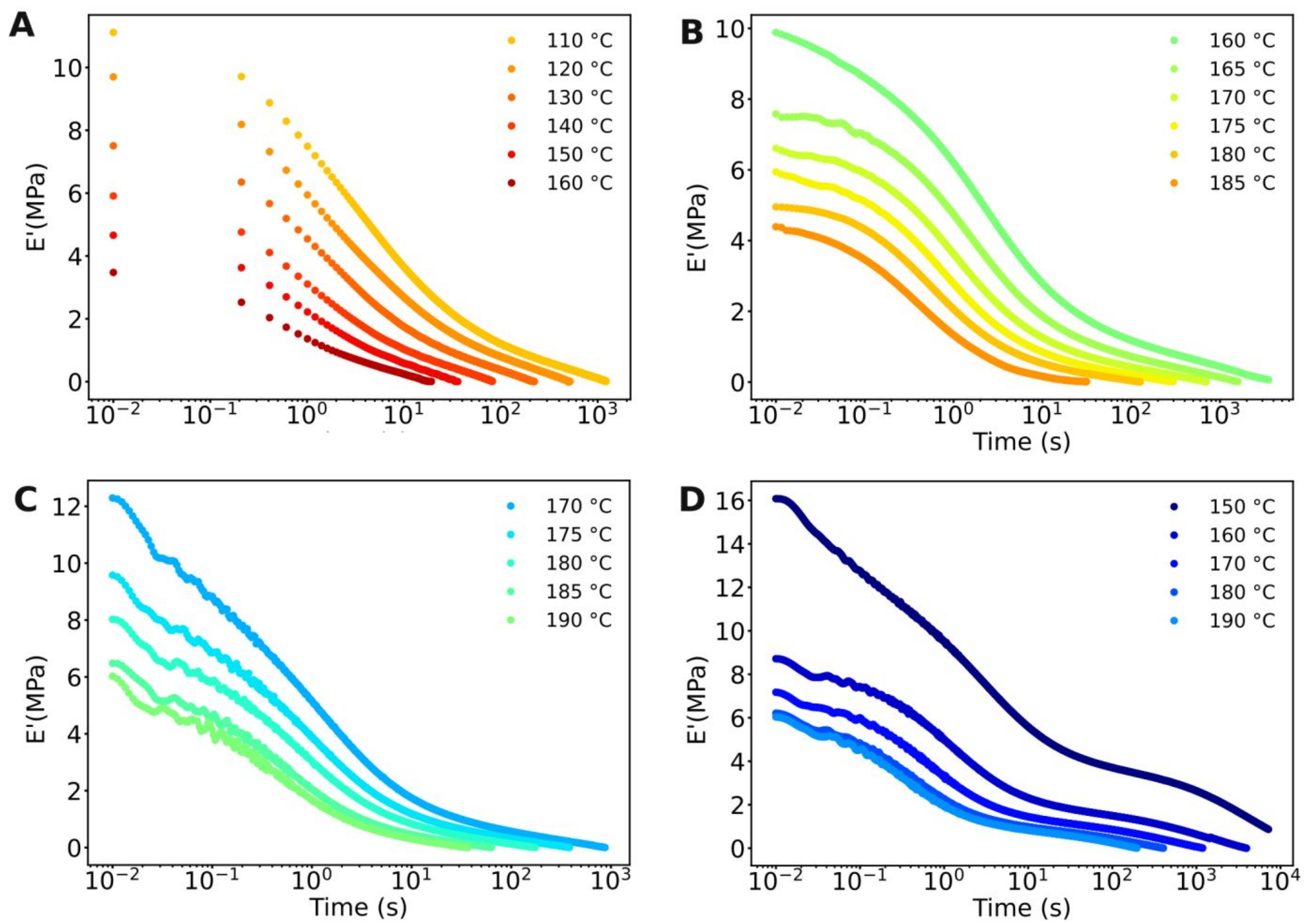

Figure S6. Non-normalized stress relaxation curves of: A) PAI-D230-0.25; B) PAI-D230-0.50; C) PAI-D230-0.75 and D) PAI-HMDA-0.25.

5.1. Continuous relaxation spectra

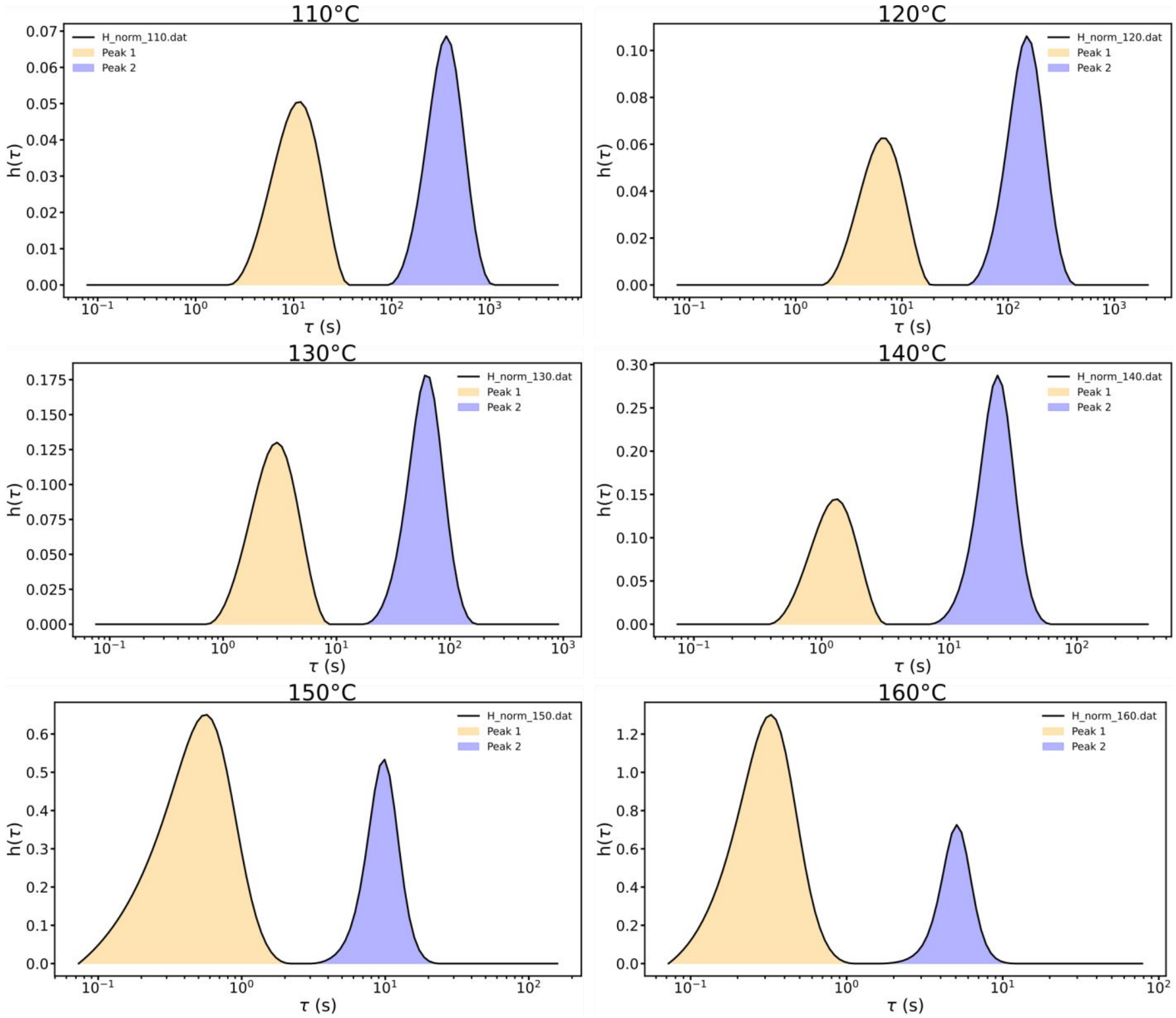

Figure S7. *h(τ)* curves, deconvolution and integration of peaks for the material PAI-D230-0.25.

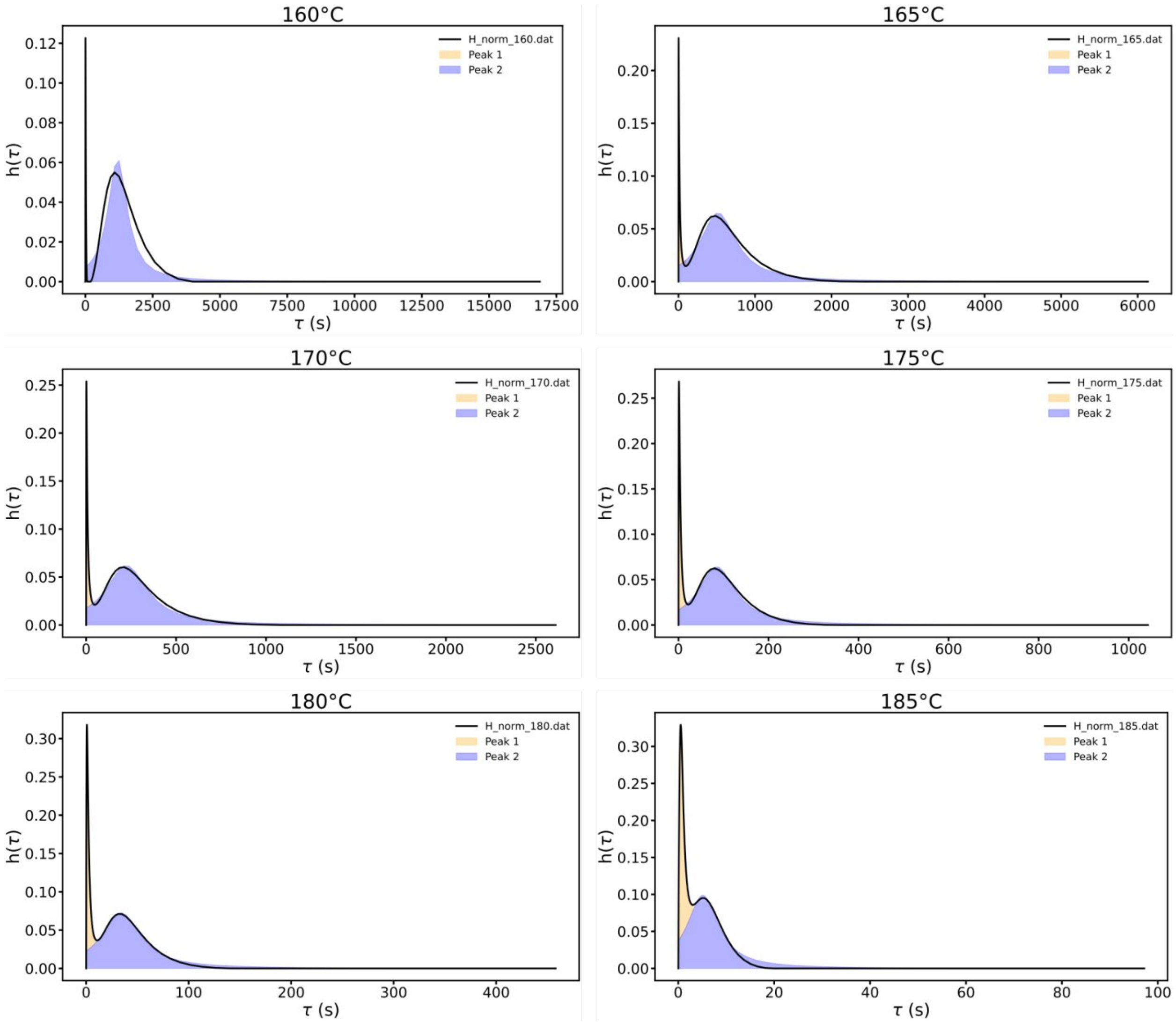

Figure S8. *h(τ)* curves, deconvolution and integration of peaks for the material PAI-D230-0.50 (x-axis is shown in linear form to better appreciate the signal shape).

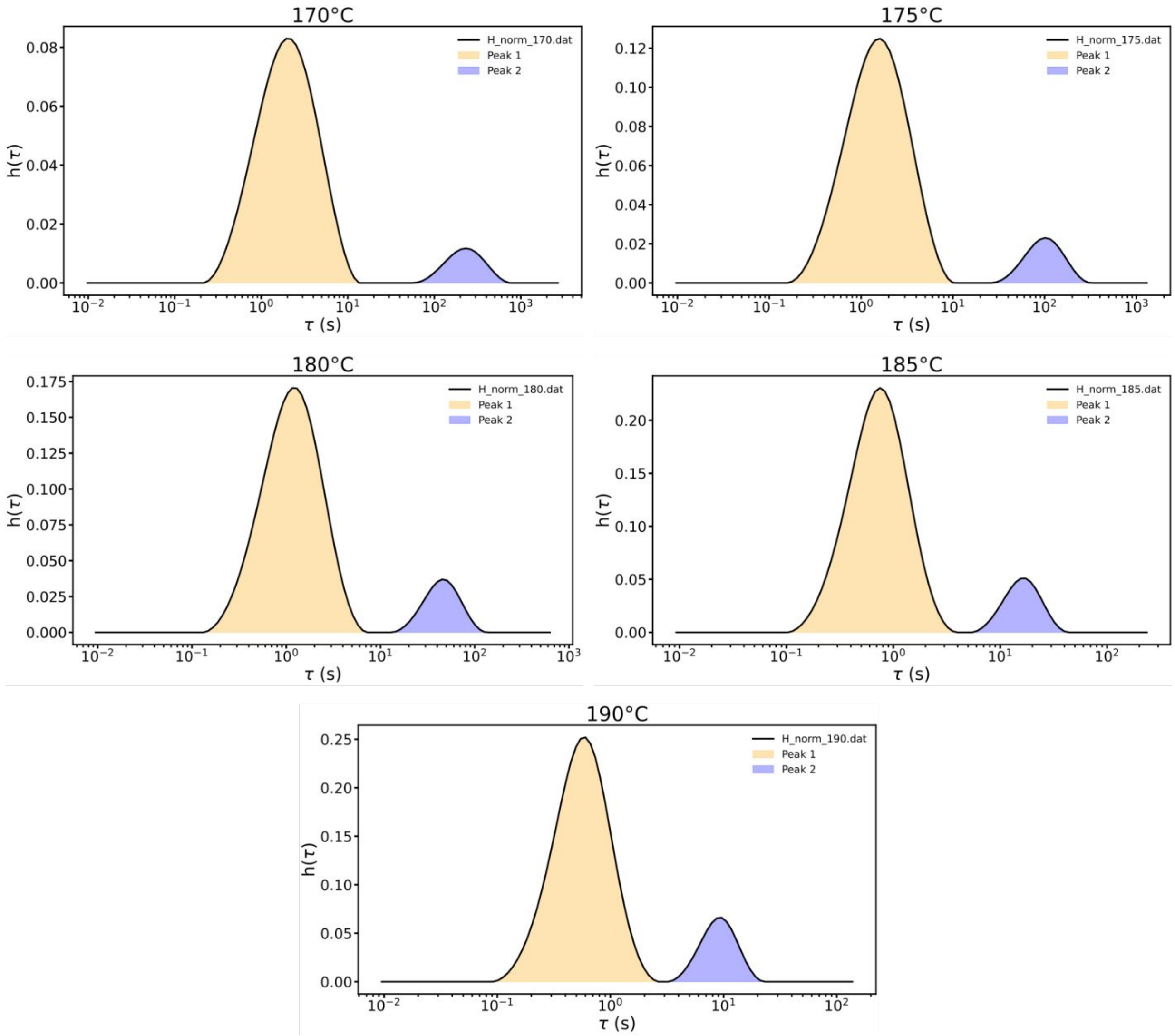

Figure S9. *h(τ)* curves, deconvolution and integration of peaks for the material PAI-D230-0.75.

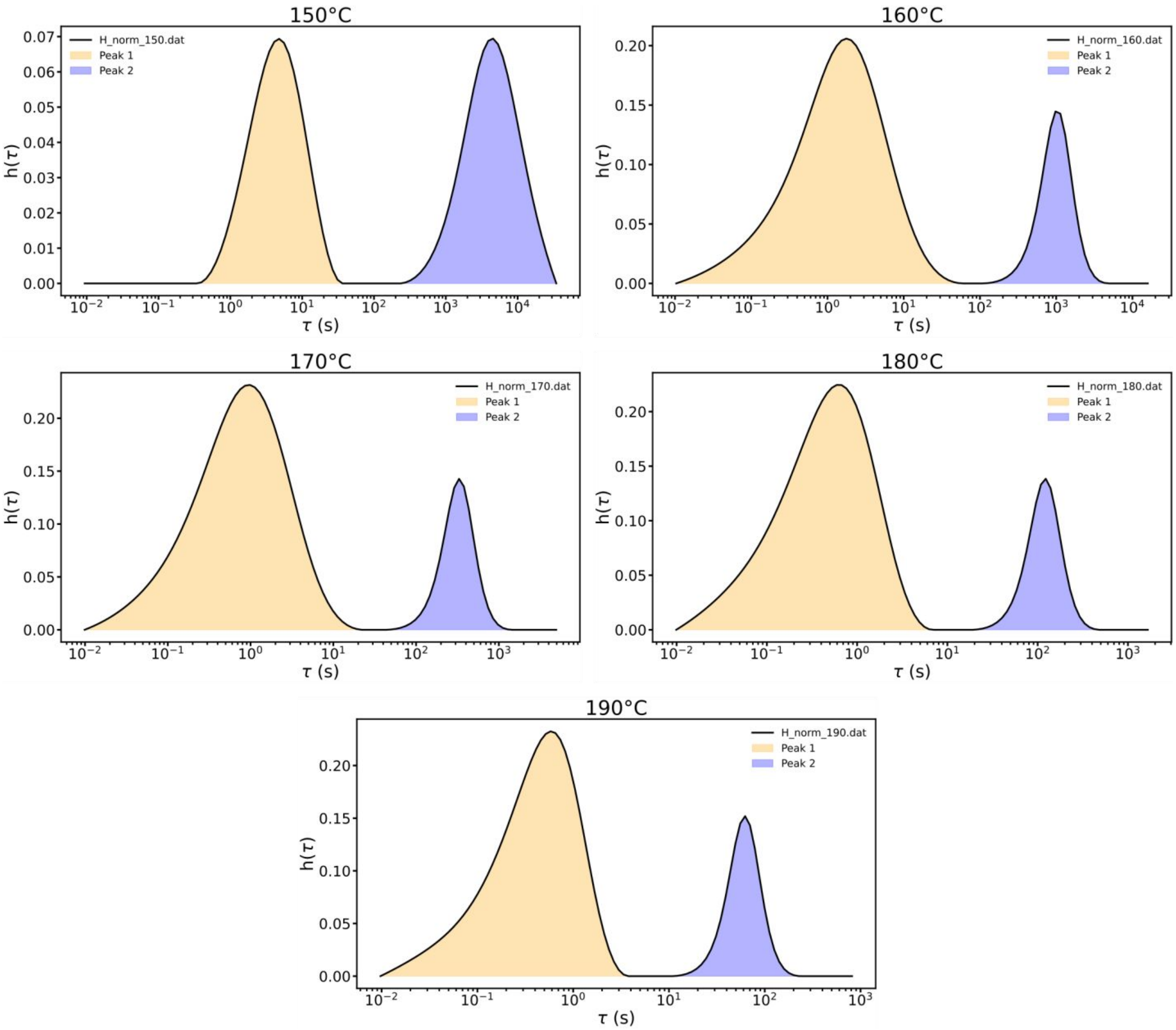

Figure S10. *h(τ)* curves, deconvolution and integration of peaks for the material PAI-HMDA-0.25.

Table S1. Characteristic relaxation times extracted from the CRS analysis.

Sample	T (°C)	τ_1^{CRS} (s)	τ_2^{CRS}(s)
**PAI-D230-0.25**	110	9	361
	120	6	149
	130	3	60
	140	1	24
	150	0.6	10
	160	0.3	5
**PAI-D230-0.50**	165	3	1082
	170	2	486
	175	1.4	211
	180	1.1	80
	185	0.7	31
	190	0.5	5
**PAI-D230-0.75**	170	1.9	203
	175	1.4	89
	180	1.2	45
	185	0.7	16
	190	0.6	9
**PAI-HMDA-0.25**	150	4.8	4556
	160	1.8	978
	170	0.9	331
	180	0.6	123
	190	0.4	62

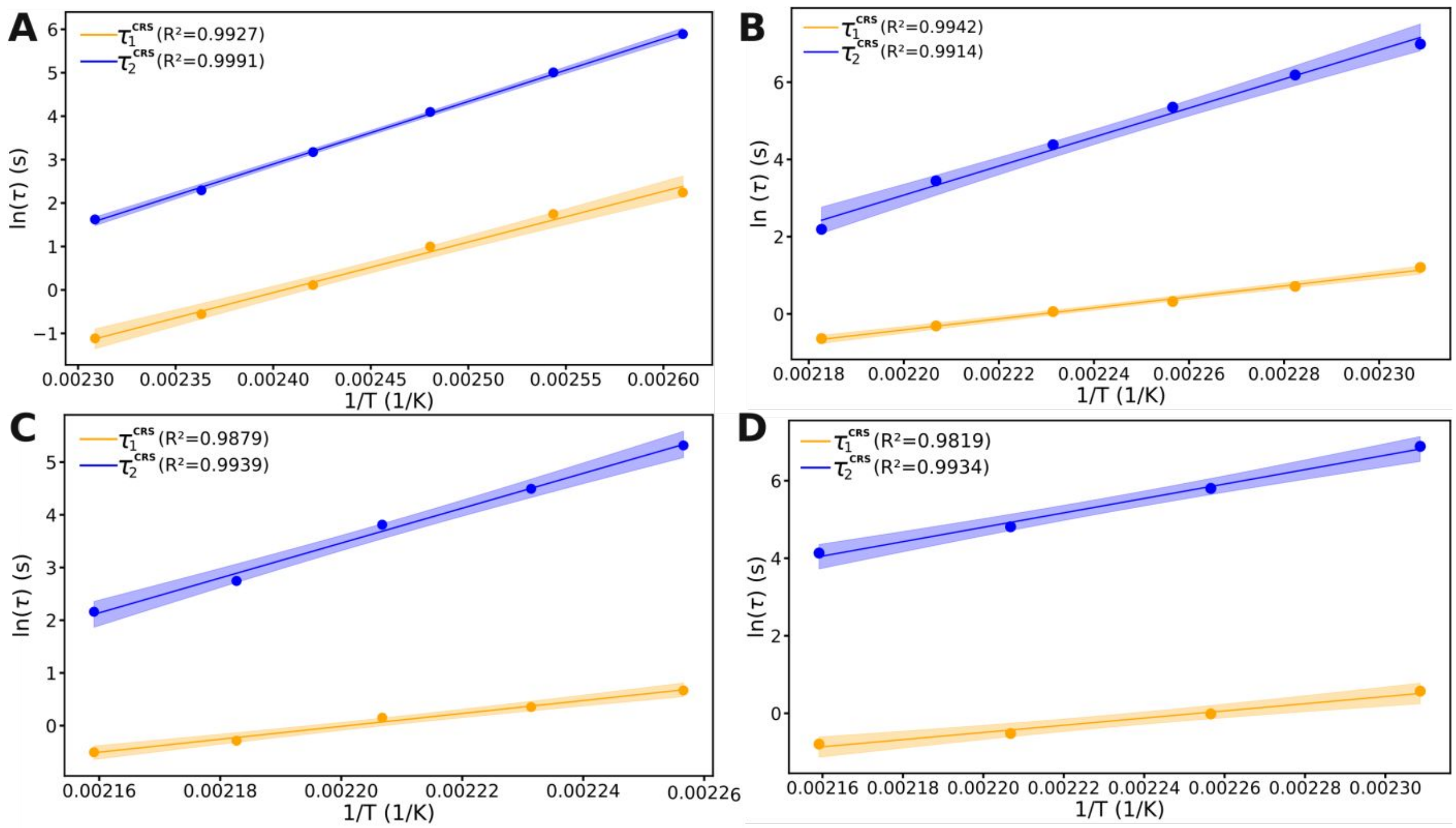

Figure S11. Arrhenius plot built from the characteristic relaxation times extracted from the CRS of: A) PAI-D230-0.25; B) PAI-D230-0.50; C) PAI-D230-0.75 and D) PAI-HMDA-0.25.

5.2. Double Kohlrausch−Williams−Watts (DKWW) model

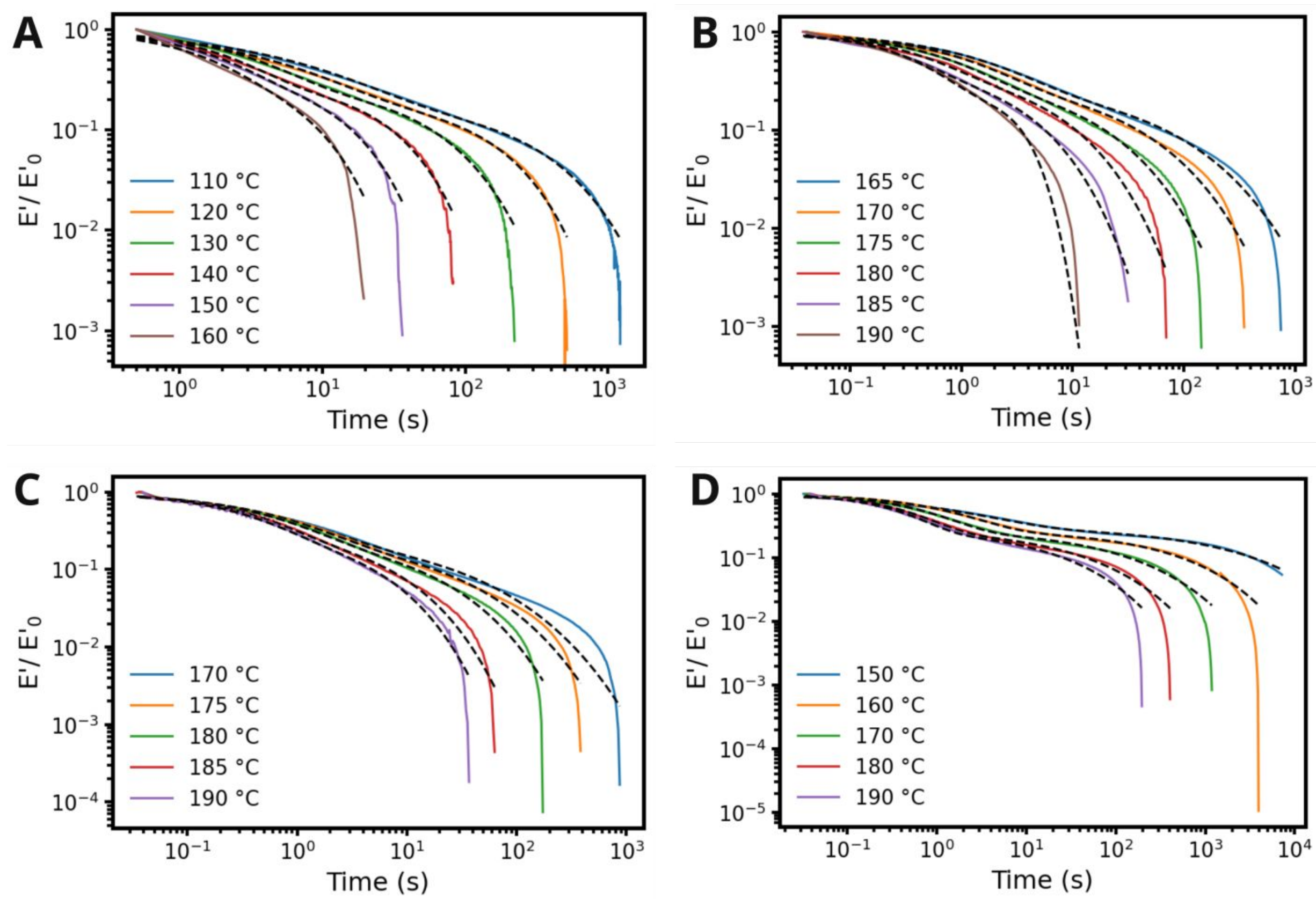

Figure S12. Stress relaxation curves and DKWW fitting of: A) PAI-D230-0.25; B) PAI-D230-0.50; C) PAI-D230-0.75 and D) PAI-HMDA-0.25.

Table S2. Average relaxation times ($\langle\tau\rangle_1$ and $\langle\tau\rangle_2$), β parameters and R^2 calculated by fitting the stress relaxation curves to DKWW model.

Sample	T (°C)	$\langle\tau_1\rangle$ (s)	β_1	$\langle\tau_2\rangle$ (s)	β_2	R^2
**PAI-D230-0.25**	110	10.0	0.582	335.2	0.800	0.996
	120	6.3	0.639	156.2	0.935	0.995
	130	3.2	0.873	58.4	0.832	0.995
	140	2.1	0.999	27.1	0.999	0.994
	150	1.7	0.999	12.3	0.999	0.988
	160	1.4	0.999	6.6	0.999	0.976
**PAI-D230-0.50**	165	2.1	0.628	108.4	0.490	0.998
	170	1.4	0.692	47.6	0.534	0.997
	175	0.9	0.737	19.7	0.568	0.998
	180	0.5	0.827	8.0	0.620	0.995
	185	0.3	0.899	3.8	0.666	0.995
	190	0.2	1.000	1.2	1.000	0.995
**PAI-D230-0.75**	170	1.1	0.581	68.1	0.428	0.993
	175	0.9	0.597	36.3	0.468	0.996
	180	0.7	0.646	18.6	0.518	0.995
	185	0.5	0.753	7.4	0.596	0.994
	190	0.4	0.746	4.8	0.617	0.995
**PAI-HMDA-0.25**	150	3.6	0.462	7131.1	0.439	0.994
	160	1.6	0.641	1060.2	0.500	0.999
	170	0.9	0.740	306.9	0.455	0.998
	180	0.5	0.820	94.9	0.459	0.997
	190	0.3	0.865	36.0	0.486	0.997

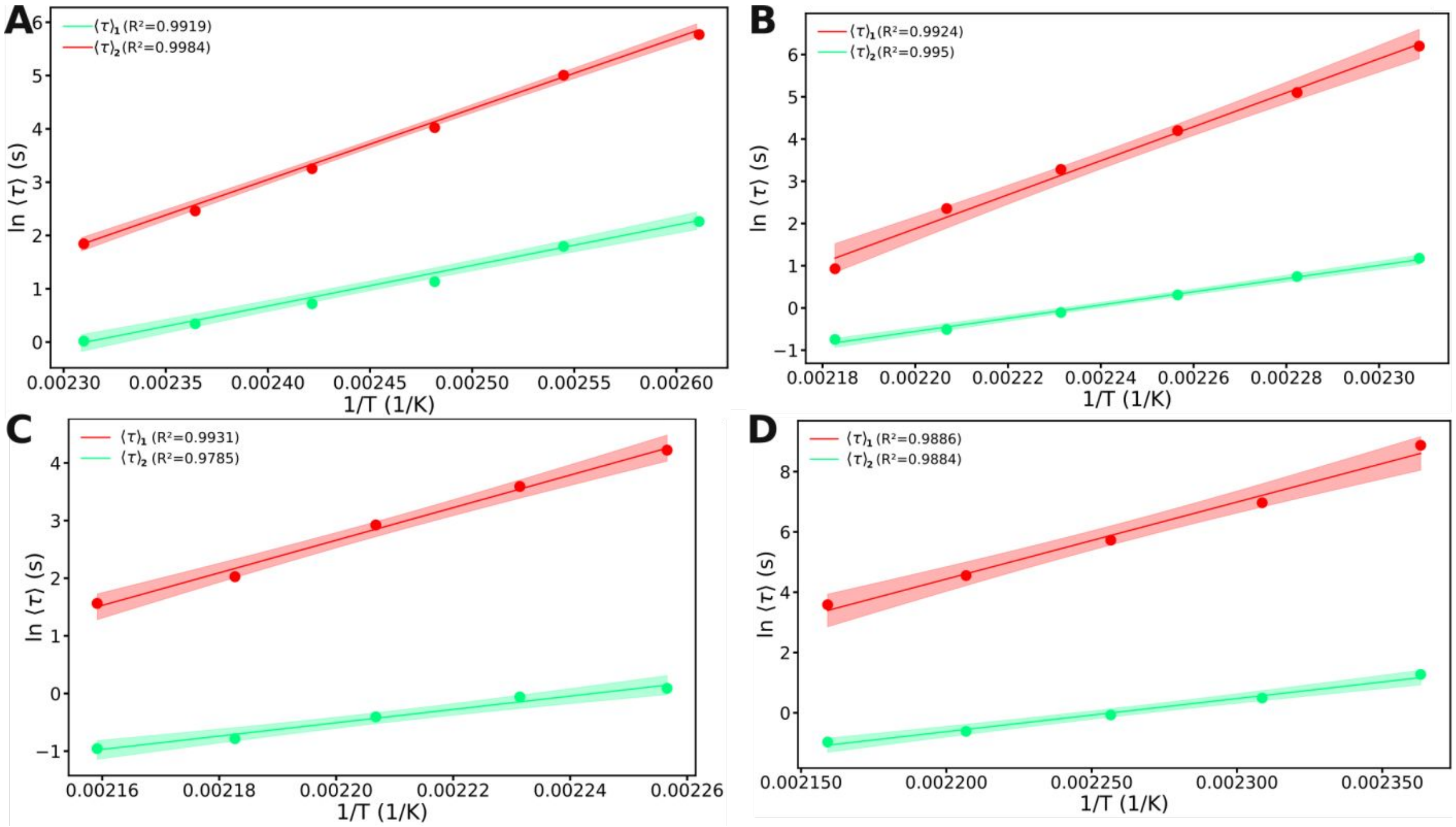

Figure S13. Arrhenius plot built from the average relaxation times extracted by fitting the DKWW model for: A) PAI-D230-0.25; B) PAI-D230-0.50; C) PAI-D230-0.75 and D) PAI-HMDA-0.25.

6. High temperature FTIR spectroscopy

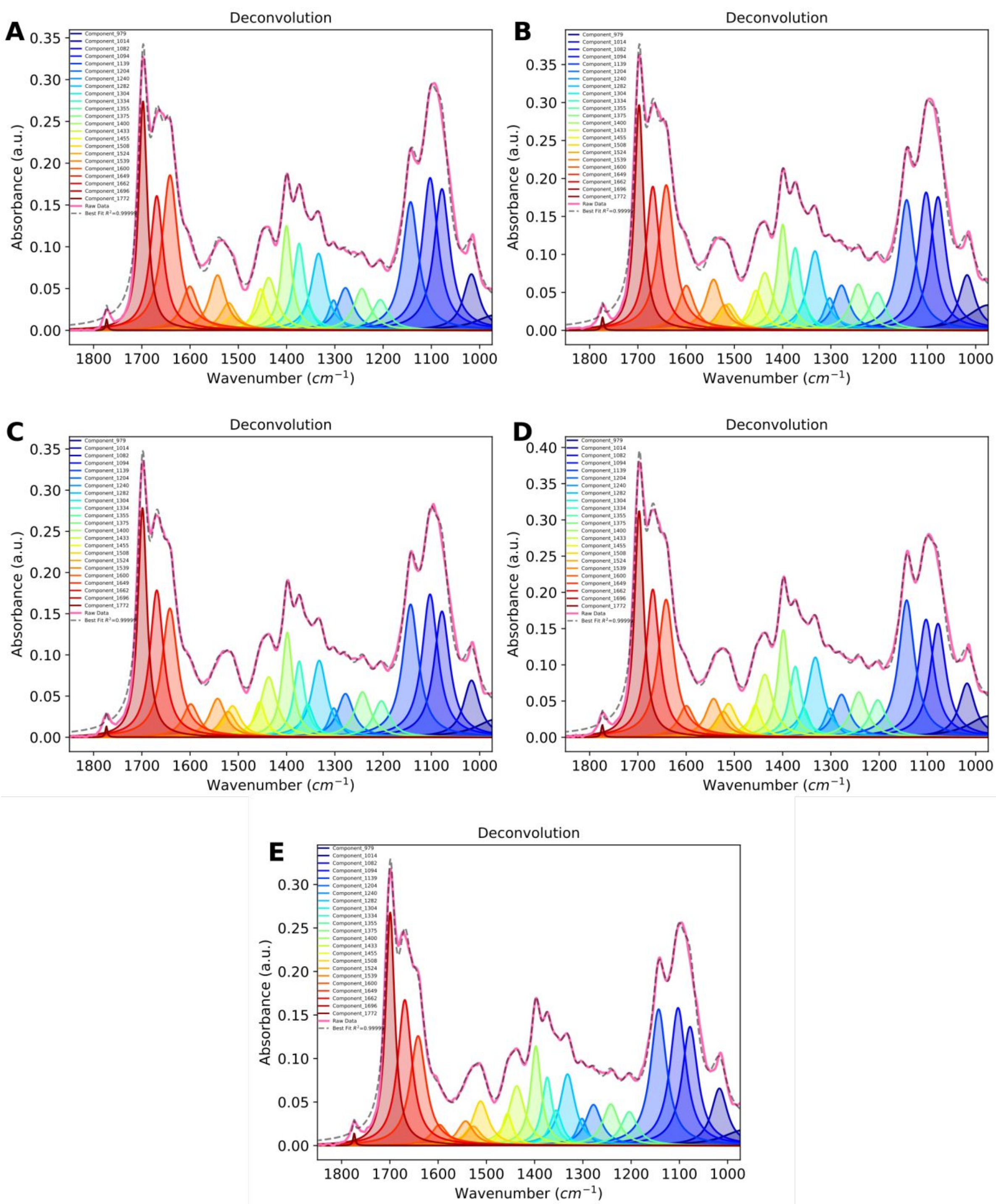

Figure S14. FTIR spectra and peak deconvolution of PAI-D230-0.25 at: A) 100 °C; B) 120 °C; C) 140 °C; D) 160 °C; E) 180 °C.

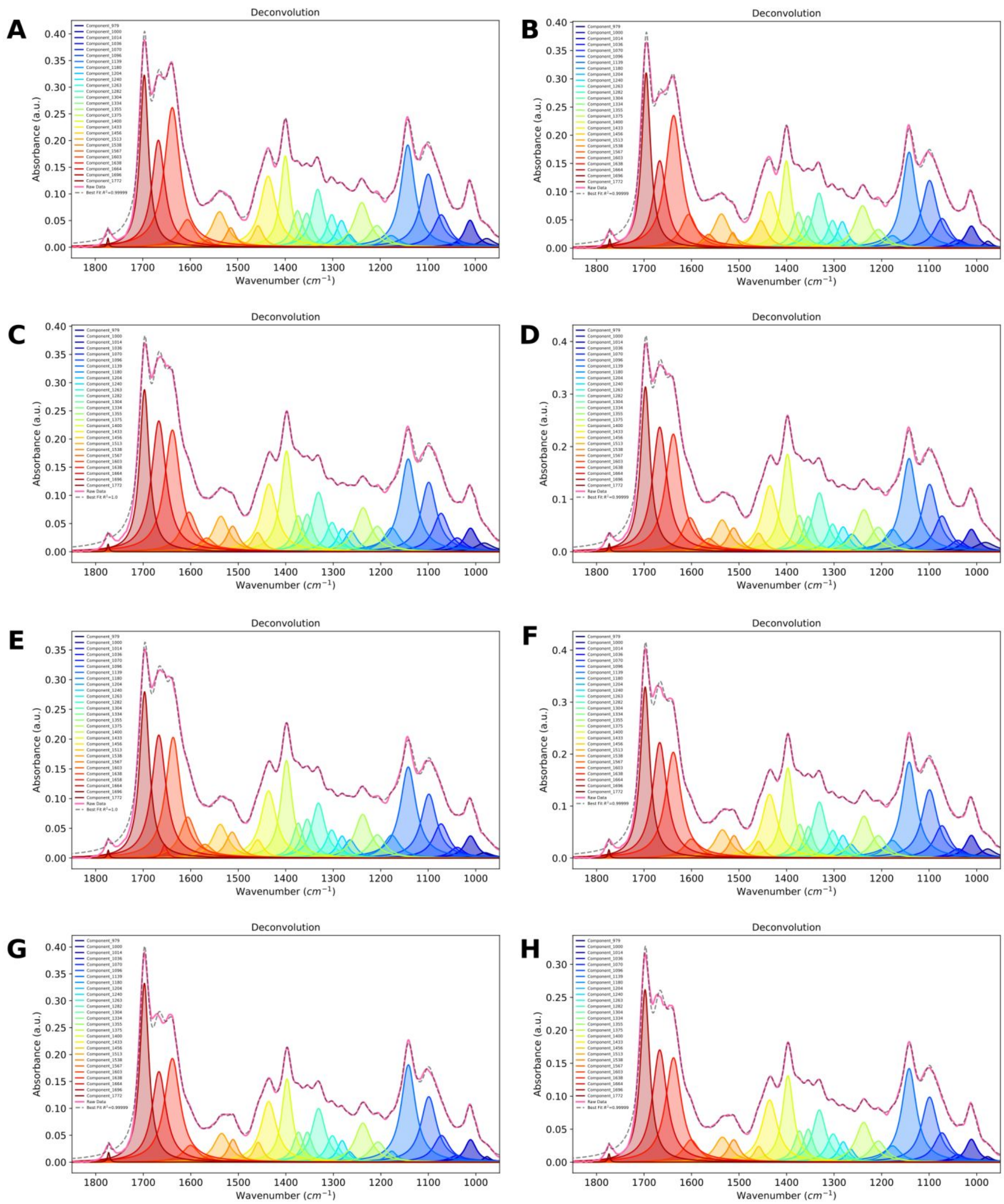

Figure S15. FTIR spectra and peak deconvolution of PAI-D230-0.50 at: A) 120 °C; B) 130 °C; C) 140 °C; D) 150 °C; E) 160 °C; F) 170 °C; G) 180 °C; H) 200 °C.

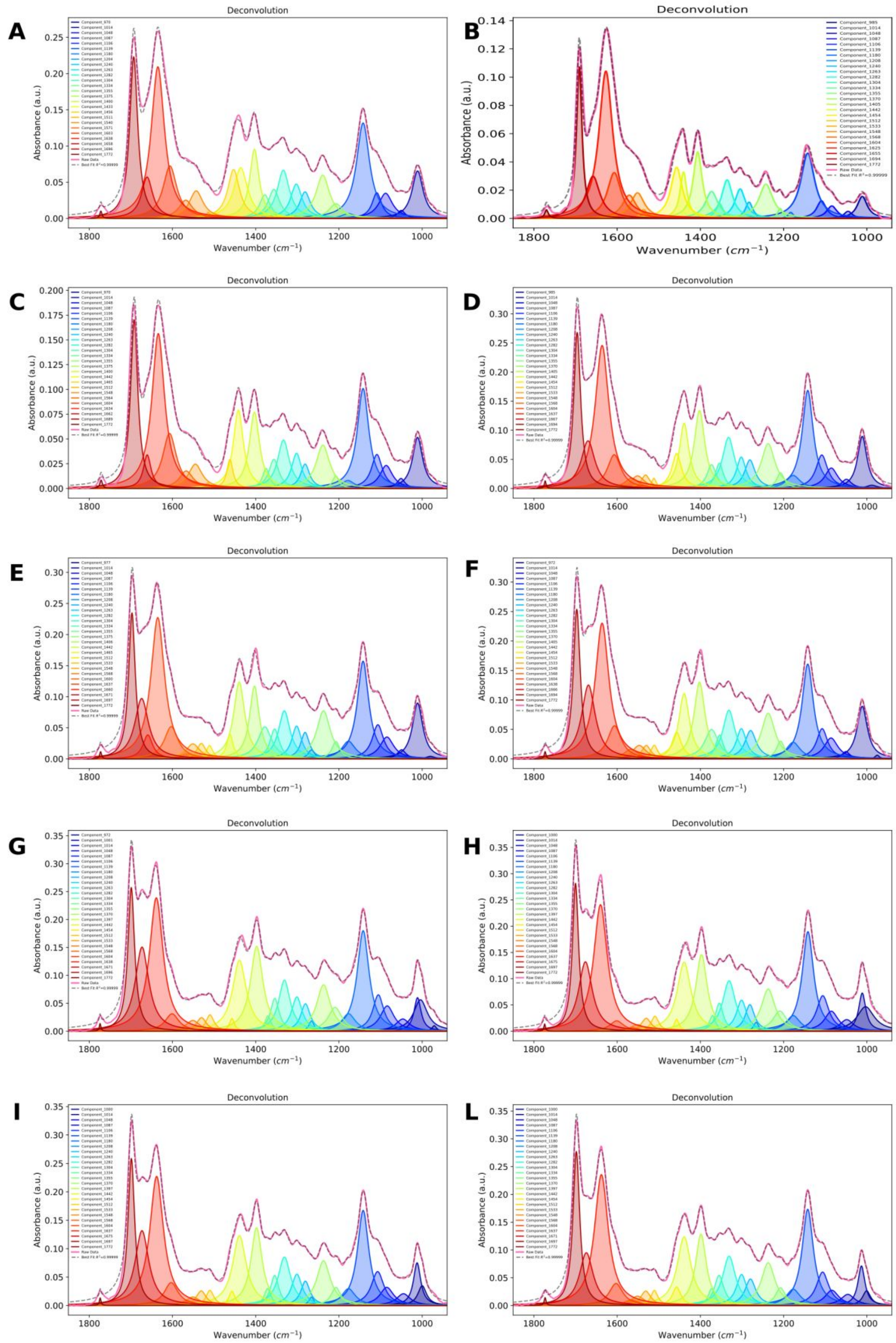

Figure S16. FTIR spectra and peak deconvolution of PAI-D230-0.75 at: A) 110 °C; B) 120 °C; C) 130 °C; D) 140 °C; E) 150 °C; F) 160 °C; G) 170 °C; H) 180 °C; I) 190 °C; L) 200 °C.

7. Creep experiments

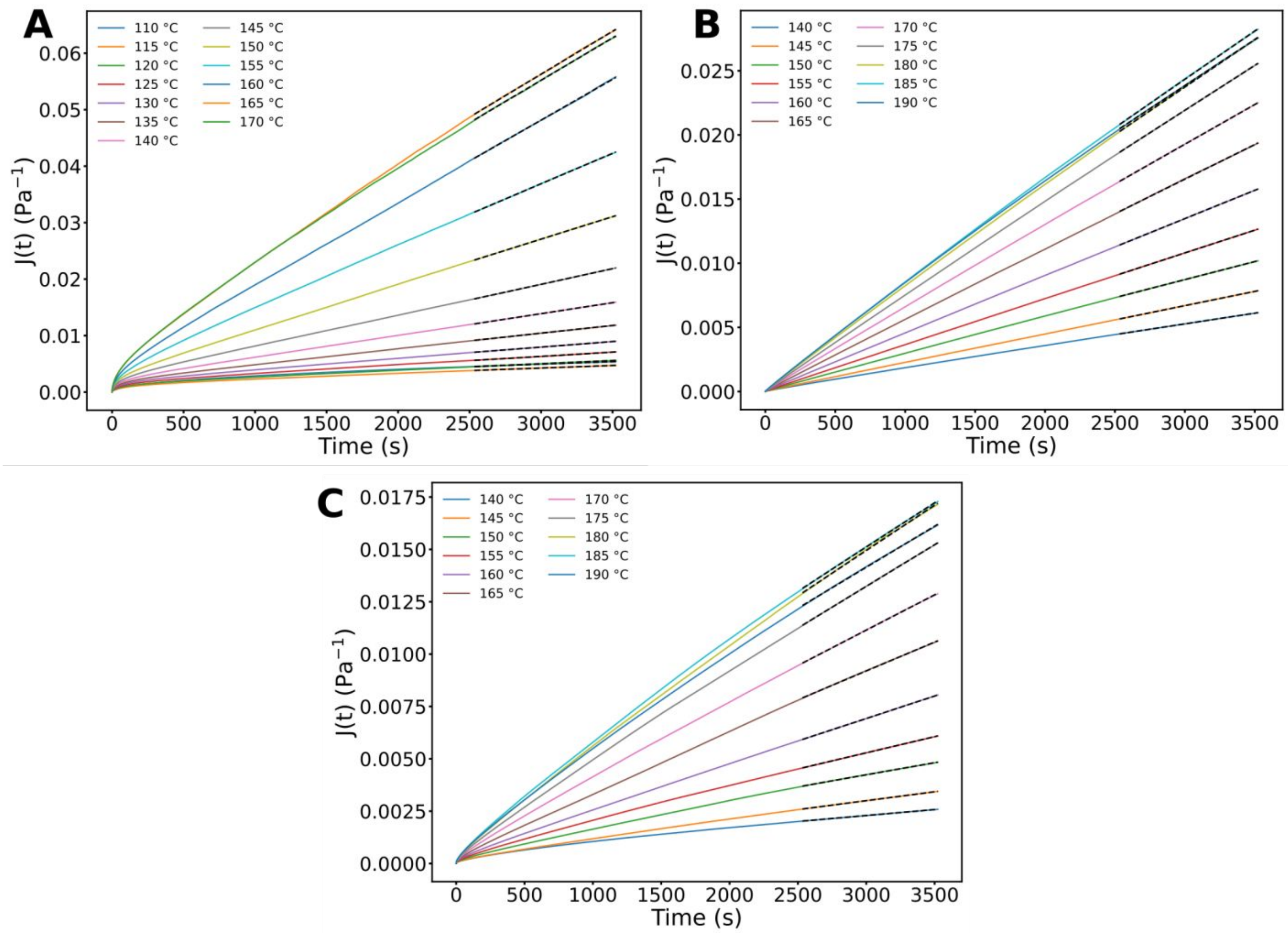

Figure S17. Creep compliance curves of experiments performed at temperatures ranging from 110 to 190 °C of: A) PAI-D230-0.25; B) PAI-D230-0.50 and C) PAI-D230-0.75 materials.

8. Lap shear tests

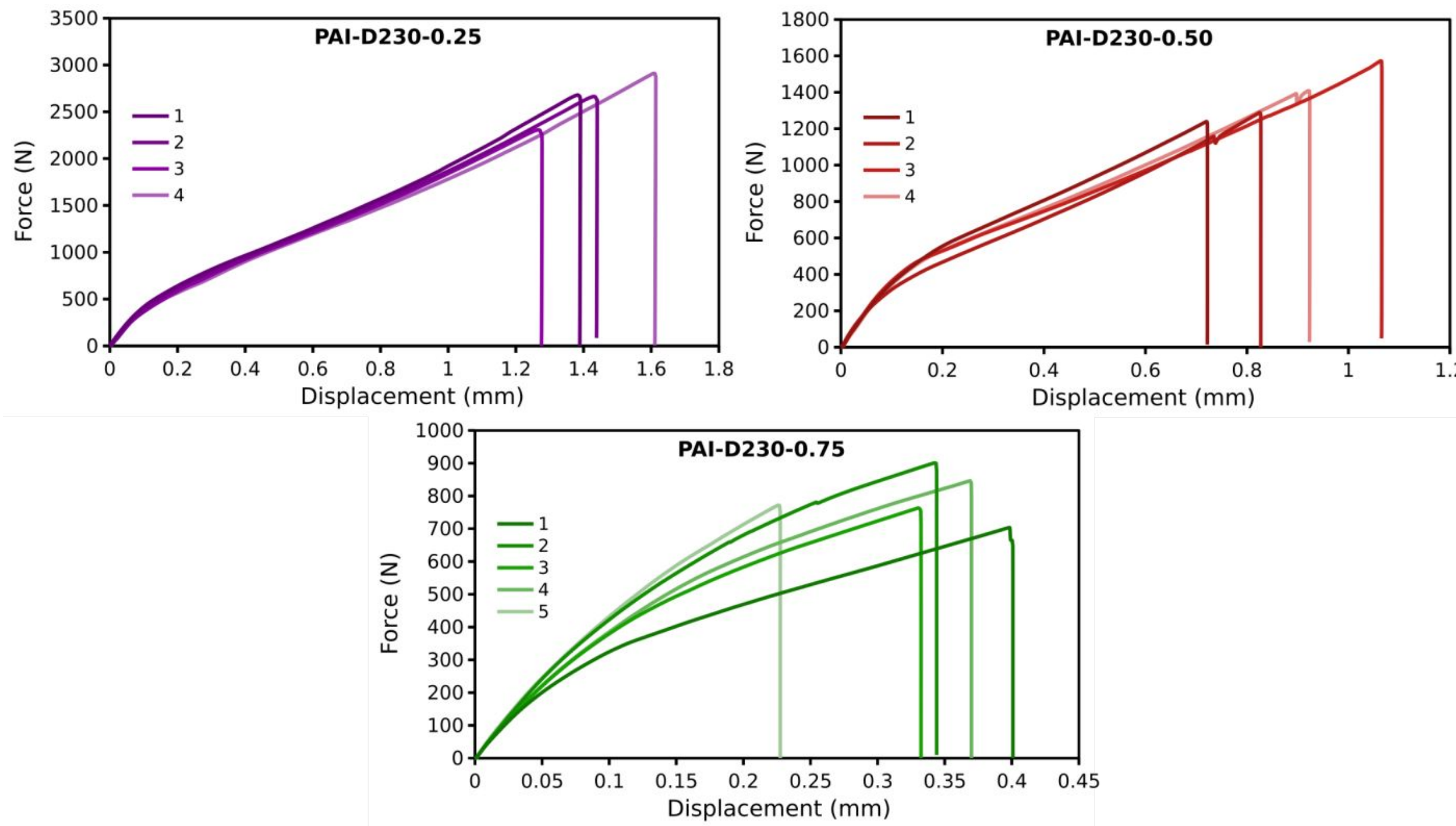

Figure S18. Force-displacement curves form lap shear tests for the different PAI-D230 materials.

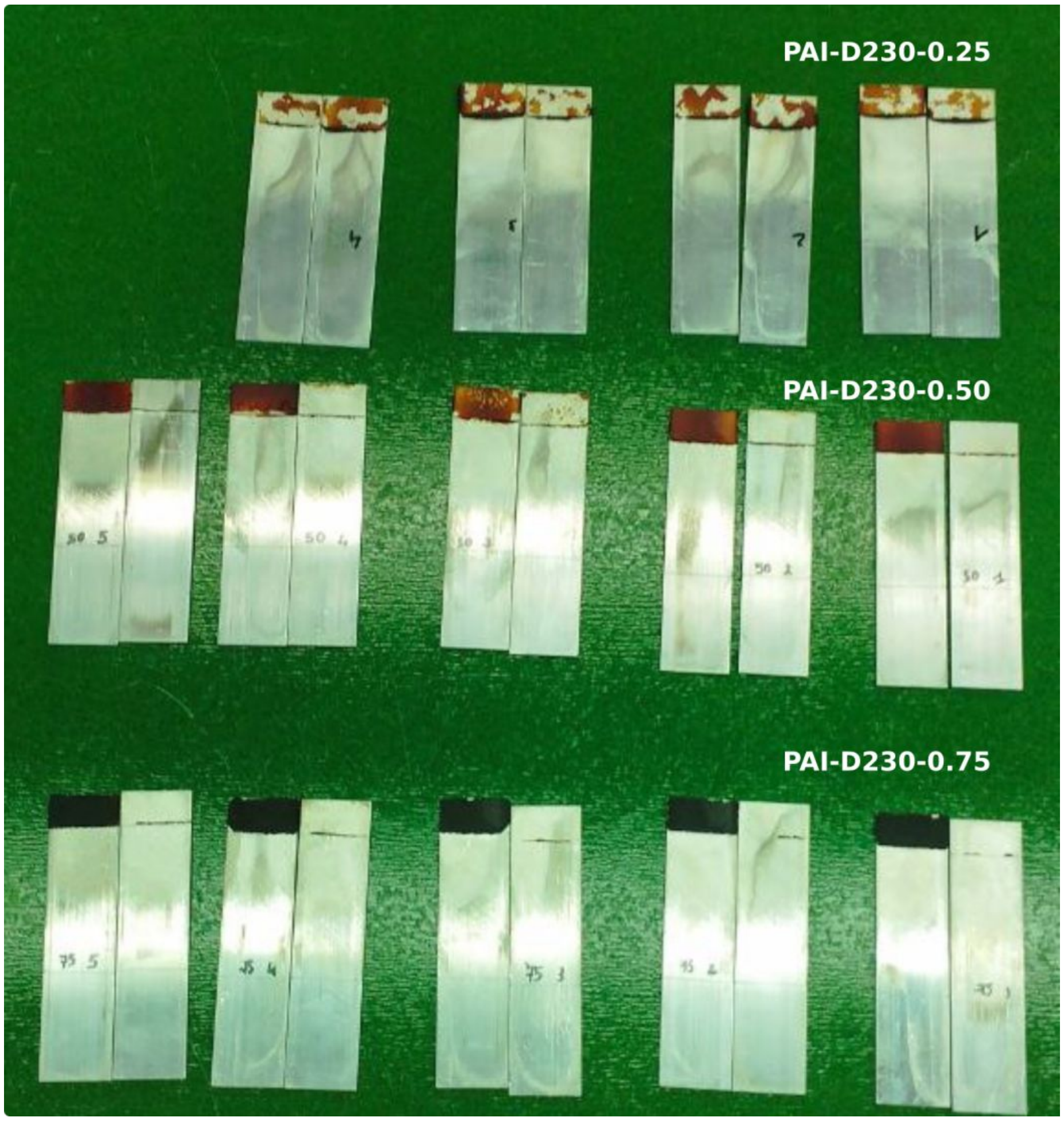

Figure S19. Pictures of the plates surface after lap-shear test of all the tested materials.

References

[1] Van Lijsebetten, F.; Spiesschaert, Y.; Winne, J.M.; Du Prez, F.E. Reprocessing of Covalent Adaptable Polyamide Networks through Internal Catalysis and Ring-Size Effects, *J. Am. Chem. Soc*. **2019**, *141*, 15277-15287.

[2] Li, M.; Li, S.; Huang, Y.; Chen, H.; Zhang, S.; Zhang, Z.; Wu, W.; Zeng, X.; Zhou, B.; Li. B. Secreted Expression of mRNA-Encoded Truncated ACE2 Variants for SARS-CoV-2 via Lipid-Like Nanoassemblies. *Adv. Mater.* **2021**, *33* (34), 2101707.
